# Robust tracking control for a quadrotor subjected to disturbances using new hyperplane-based fast Terminal Sliding Mode

**DOI:** 10.1371/journal.pone.0283195

**Published:** 2023-04-24

**Authors:** Moussa Labbadi, Jamshed Iqbal, Mohamed Djemai, Yassine Boukal, Yassine Bouteraa

**Affiliations:** 1 Univ. Grenoble Alpes, CNRS, Grenoble INP, GIPSA-lab, Grenoble, France; 2 School of Computer Science, Faculty of Science and Engineering, University of Hull, Hull, United Kingdom; 3 Aeronautics, Space & Defense Division, ALTRAN, Blagnac, France; 4 College of Computer Engineering and Sciences, Prince Sattam bin Abdulaziz University, Al-Kharj, Saudi Arabia; 5 Control and Energy Management Laboratory (CEM Lab.), Ecole Nationale d Ingenieurs de Sfax (ENIS) & Institut Superieur de Biotechnologie de Sfax (ISBS), University of Sfax, Sfax, Tunisia; Beijing University of Posts and Telecommunications, CHINA

## Abstract

This paper presents a finite-time approach for tracking control of a quadrotor system subjected to external disturbances and model uncertainties. The proposed approach offers a preassigned performance guarantee. Firstly, integral terminal sliding manifolds and nonsingular terminal sliding manifolds are considered to produce the new hyperplane sliding variables for both position and attitude of a quadrotor. The designed hyperplane sliding variables guaranteed a finite-time convergence. The objective is to develop a finite-time control scheme for a disturbed quadrotor to follow a predefined trajectory based on a nonlinear sliding mode controller. The main contribution of this paper is to design a hyperplane-based nonlinear sliding mode control strategy for a quadrotor subjected to disturbances. A concept of robust controllers for a quadrotor is presented based on Lyapunov theory, which proves finite-time stability of the proposed control technique. Numerical simulations with two different scenarios verify the accuracy of the proposed hyperplane-based sliding mode control approach. The simulations study also included a comparison with another nonlinear controller. Results demonstrated overperformance of the proposed control strategy.

## 1 Introduction

Quadrotor unmanned aerial vehicles (UAVs) have been used in a variety of applications, including precision takeoff, tactical reconnaissance, management and rescue missions, environmental protection, courier/delivery, surveillance, and reconnaissance operations; [1, 2]. The control of the quadrotor can be achieved by precisely following a particular trajectory. The quadrotor system is highly nonlinear and inherently unstable by nature [3, 4]. In addition, the highly coupled dynamics of a quadrotor makes control design more difficult. Furthermore, during indoor/outdoor flight, the quadrotor system is subject to nonlinearities from multiple sources such as model uncertainties, external disturbances, and unmodeled dynamics, which reduces the accuracy of the quadrotor UAV tracking control.

Therefore, the design of a robust tracking controller is the key to overcoming these problems and enabling the quadrotor to track a predefined trajectory. Adaptive backstepping was introduced by [5, 6] to improve the tracking performance of a quadrotor. The authors of [7–10] proposed a fractional order (FO) sliding mode control (SMC) technique to improve the transient performance of a quadrotor system. The authors of [11] proposed the self-triggered SMC for a quadrotor under disturbances. In recent years, different SMC techniques have been devoted to tracking control [12, 13]. In this paper, a finite time control is proposed for the position and attitude of a quadrotor under disturbances. There are some other nonlinear and intelligent control methods like fuzzy logic control [14], which uses min-max rules that are not robust and pose difficulties in proving its analytical stability. Similarly, backstepping can be used to track the desired trajectory of a quadrotor, however, convergence in finite time is not assured by this control method.

In terms of ease of implementation and robustness against uncertainties and external disturbances, the SMC is a potent tool for complex nonlinear systems. In our previous work [15], we used a nonlinear manifold with non-singular terminal sliding mode (NTSM) for a quadrotor. The disadvantage of the SMC is the occurrence of chattering in system inputs [16]. Several solutions have been proposed in the literature to resolve this chattering problem, including the super twisting integral SMC [17], high-order SMC [18], and the finite-time control methods including, fast terminal SMC (FTSMC) algorithms [12]. The TSMC’s biggest flaw is its one-of-a-kind problem. A new continuous integral of the sign of the error is proposed in [19] to address this problem while maintaining robustness. Also used was a decent variety of the terminal SMC (TSMC) method known as nonsingular TSMC (NTSMC) [15]. As a result of integrating the benefits of ITSMC and NTSMC, this research proposes a new hyperplane sliding mode technique for quadrotor systems subjected to external disturbances.

In [20], an improved integral of signum error control technique is investigated for the robust tracker design of quadrotor system under disturbances. In [21], the super-twisting algorithm and adaptive dynamic programming techniques have been combined for the tracking control problem of quadrotor subjected to complex disturbances. In order to estimate the external disturbances, an adaptive TSM disturbance observer is proposed. The work presented in [22] combined adaptive super twisting and nonsingular TSMC for quadrotor in the presence of bounded disturbances. Online control laws are designed to estimate exactly the upper bound of disturbances. The authors of [13] proposed a novel FO fast integral TSMC technique for position/attitude of a quadrotor to enhance the tracking performance against external disturbances. To achieve a finite-time tracking control of a quadrotor under actuators, disturbances, and input saturation, the authors in [23] proposed a neural network based on a fault tolerance control approach. The authors of [24] proposed a fixed-time convergence and disturbance rejection control approaches for a quadrotor attitude. The work developed in [25] combined a fixed nonsingular TSMC and observer for a robust control tracking of uncertain quadrotor under uncertainties. In order to stabilize an uncertain quadrotor and to make it to track a predefined flight trajectory, two PD control techniques, and adaptive fuzzy TSMC are proposed in [26]. In [27], a new observer-based control approach is proposed for controlling a quadrotor under disturbances and noisy measurements. The authors of [28] investigated to present new finite-time control and fixed time prescribed performance for a quadrotor system. In order to reduce the chattering problem, the paper [29] presented an aperiodic signal updating for a quadrotor under external disturbances and uncertainties. The authors of [30] proposed an adaptive finite-time control for a quadrotor using backstepping and global sliding mode controllers. In [31], barrier function and nonsingular terminal SMC are proposed for a quadrotor. Event-triggered fractional-order SMC approach was proposed for UAV under disturbances. In [32], a conditional integrator SMC was developed for a quadrotor. An observer based rotor failure compensation for a quadrotor was proposed in [33]. In [34], a hybrid controller based on backstepping and integral SMC was proposed for a quadrotor. In [35], an observer-based backstepping control was proposed for a uas.

In this paper, a robust integral non-singular hyperplane SMC (INH-SMC) scheme is designed to control the disturbed quadrotor and ensures accurate tracking under the effect of disturbances. For the attitude and position subsystems, novel integral-type hyperplane-based sliding manifolds are designed. The proposed manifolds for quadrotor system are designed by combining integral-type TSMC and nonsingular TSMC to obtain robust, accurate tracking performance, and fast convergence of the state variables. The result input signals are integrated to achieve continuous controllers, which reduces the chattering phenomenon. The proposed control scheme addressed and rejected the disturbances. Contributions of this research paper can be highlighted as follows:

- Integral-type sliding and nonsingular terminal sliding manifolds are combined and applied to attitude and position of a quadrotor, which offers high tracking accuracy and faster convergence, reduces the steady-state error, and demonstrates stronger robustness against disturbances.
- Switching laws are proposed to deal with the upper bound of the disturbances that affects the dynamics.
- The proposed control scheme is applied to quadrotor dynamics in the presence of disturbances and confirmed its superiority compared to super twisting algorithms by simulation results.

The rest parts of the present paper are structured as follows. The formulation problem is given in Section II. The proposed control scheme and its stability are presented in Section III. The results are provided in Section IV. Finally, conclusions are presented in Section V.

## 2 Model of flight dynamics

**Fig 1:**
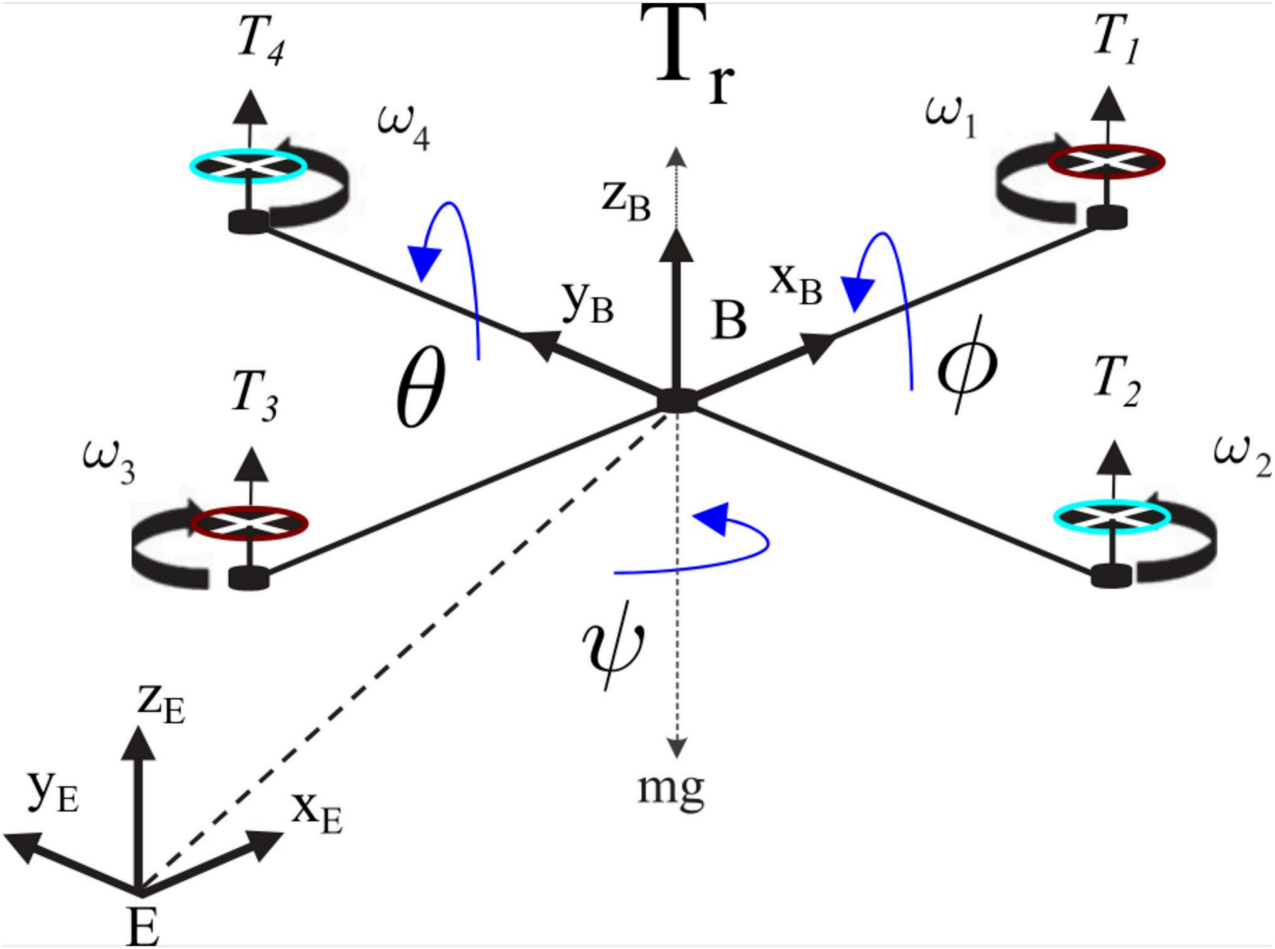
Quadrotor configuration.

In this section, a modeling system of a quadrotor is presented. As depicted in Fig 1, two frames are defined: an inertial reference frame *E* = {*O*_*e*_, *X*_*e*_, *Y*_*e*_, *Z*_*e*_} and a body-fixed frame *B* = {*O*_*b*_, *X*_*b*_, *Y*_*b*_, *Z*_*b*_}.

Let’s define the Euler angles related to an inertial frame and the angle velocities respectively by $X_{\omega }\left(t\right)={\left[\phi \left(t\right),\;\theta \left(t\right),\;\psi \left(t\right)\right]}^{T}$ and ${\dot{X}}_{\omega }\left(t\right)={\left[\dot{\phi }\left(t\right),\;\dot{\theta }\left(t\right),\;\dot{\psi }\left(t\right)\right]}^{T}$. The linear velocity can be defined $V\left(t\right)={\left[p\left(t\right),\;q\left(t\right),\;r\left(t\right)\right]}^{T}$. Let’s introduce the position and linear velocity in the earth-frame respectively by $X_{ϒ}\left(t\right)={\left[x\left(t\right),\;y\left(t\right),\;z\left(t\right)\right]}^{T}$ and $V={\left[u\left(t\right),\;v\left(t\right),\;w\left(t\right)\right]}^{T}$. In order to obtain the dynamic model of the quadrotor, following assumptions are considered as in [15]:

**Assumption 1**. *The construction of quadrotor is symmetrical and rigid*.

**Assumption 2**. *The yaw, roll/pitch angles are limited respectively by* $\left(-\frac{\pi }{2},\frac{\pi }{2}\right)$ *and* (−*π*, *π*).

Using the Newton-Euler laws, the quadrotor model can be presented in the following equation.

$${\dot{X}}_{ϒ}\left(t\right)=V(t)$$ (1a)

$$\omega (t)=R_{q}X_{\omega }\left(t\right)$$ (1b)

$$I\omega (t)=-\omega \left(t\right)\times \left(I\omega \left(t\right)\right)+L_{res},$$ (1c)

in which, $I=diag\left(I_{x},\;I_{y},\;I_{z}\right)\in R^{3x3}$ is a symmetric positive matrix that represents the inertia of the quadrotor axes. The notation $R_{q}$ is the rotation velocities matrix which is given as:

$$\begin{array}{c} R_{q}=[\begin{array}{ccc} 1 & 0 & -\text{sin}(\theta (t))\\ 0 & \text{sin}(\phi (t)) & \text{cos}(\theta (t))\;\text{sin}(\phi (t))\\ 0 & -\text{sin}(\phi (t)) & \text{cos}(\theta (t))\;\text{cos}(\phi (t)) \end{array}] \end{array}$$ (2)

$L_{res}$ is the contributed moment torque in the quadrotor center, which is written as:

$$\begin{array}{c} L_{res}=L-L_{G}-L_{D} \end{array}$$ (3)

where L is the torque provided by four rotors quadrotor.

$$\begin{array}{c} L=[\begin{array}{c} u_{2}\\ u_{3}\\ u_{4} \end{array}]=[\begin{array}{cccc} 0 & -\rho_{y}d & 0 & -\rho_{y}d\\ -\rho_{y}d & 0 & \rho_{y}d & 0\\ -\rho_{z} & \rho_{z} & -\rho_{z} & \rho_{z} \end{array}][\begin{array}{c} \omega_{1}^{2}\\ \omega_{2}^{2}\\ \omega_{3}^{2}\\ \omega_{4}^{2} \end{array}] \end{array}$$ (4)

with *u*_2_, *u*_3_, *u*_4_ denote the quadrotor torques. *ρ*_*y*_ is a positive value representing the lift constant, *d* represents the distance between the quadrotor mass center and rotor, and *ρ*_*z*_ is the drag factor.

The gyroscopic effect can be expressed as $L_{G}=\sum_{i=1}^{4}J_{r}\left(\omega \times e_{3}\right){\left(-1\right)}^{+1}+\omega_{i}$, in which *j*_*r*_ denotes the inertia of the rotor blade, *ω* is the rotor speed, and *e*_3_ = [0, 0, 1]^*T*^. The $L_{D}$ is recognized as aerodynamic friction torques which is defined as $L_{D}=diag\left(K_{1},\;K_{2},\;K_{3}\right)$, while *K*_1_, *K*_2_, *K*_3_ are positive aerodynamic drag coefficients. The mathematical model of the QUAV in the presence of disturbances can be presented as follows:

$$\begin{array}{c} \begin{array}{cc} \ddot{\phi }\left(t\right) & =M_{1}\dot{\theta }\left(t\right)\dot{\psi }\left(t\right)+M_{2}\dot{\theta }\left(t\right)+M_{3}{\dot{\phi }}^{2}\left(t\right)+N_{1}u_{4}+D_{\phi }\left(t\right)\\ \ddot{\theta }\left(t\right) & =M_{4}\dot{\phi }\left(t\right)\dot{\psi }\left(t\right)+M_{5}\dot{\phi }\left(t\right)+M_{6}{\dot{\theta }}^{2}\left(t\right)+N_{2}u_{3}+D_{\theta }\left(t\right)\\ \ddot{\psi }\left(t\right) & =M_{7}\dot{\phi }\left(t\right)\dot{\theta }\left(t\right)+M_{8}{\dot{\psi }}^{2}\left(t\right)+N_{3}u_{4}+D_{\psi }\left(t\right)\\ \ddot{x}\left(t\right) & =M_{9}\dot{x}\left(t\right)+\frac{1}{m}\left(\text{cos}\;\phi (t)\;\text{sin}\;\theta (t)\;\text{cos}\;\psi (t)+\text{sin}\;\phi (t)\;\text{sin}\;\psi (t)\right)u_{1}+D_{x}\left(t\right)\\ \ddot{y}\left(t\right) & =M_{10}\dot{y(t)}+\frac{1}{m}\left(\text{cos}\;\phi (t)\;\text{sin}\;\theta (t)\;\text{sin}\;\psi (t)-\text{sin}\;\phi (t)\;\text{cos}\;\psi (t)\right)u_{1}+D_{y}\left(t\right)\\ \ddot{z}\left(t\right) & =M_{11}\dot{z}\left(t\right)-g+\frac{1}{m}\left(\text{cos}\;\phi (t)\;\text{cos}\;\theta (t)\right)u_{1}+D_{z}\left(t\right) \end{array} \end{array}$$ (5)

with: $M_{1}=\frac{\left(I_{y}-I_{z}\right)}{I_{x}}$, $M_{2}=\frac{-\omega_{r}J_{r}}{I_{x}}$, $M_{3}=\frac{-K_{1}}{I_{x}}$, $M_{4}=\frac{\left(I_{z}-I_{x}\right)}{I_{y}}$, $M_{5}=\frac{\omega_{r}J_{r}}{I_{y}}$, $M_{6}=\frac{-K_{2}}{I_{y}}$, $M_{7}=\frac{\left(I_{x}-I_{y}\right)}{I_{z}}$, $M_{8}=\frac{-K_{3}}{I_{z}}$, $M_{9}=\frac{-K_{5}}{m}$, $M_{10}=\frac{-K_{4}}{m}$, $M_{11}=-\frac{K_{6}}{m}$, $N_{1}=\frac{d}{I_{x}}$, $N_{2}=\frac{d}{I_{y}}$, $N_{3}=\frac{1}{I_{z}}$ and *ω*_*r*_ = *ω*_1_ − *ω*_2_ + *ω*_3_ − *ω*_4_. $D_{i}\left(t\right)={\left[D_{x}\left(t\right),D_{y}\left(t\right),D_{z}\left(t\right),D_{\theta }\left(t\right),D_{\theta }\left(t\right),D_{\psi }\left(t\right)\right]}^{T}$ is time-varying bounded disturbance. The underactuated problem is solved by creating the following virtual control inputs.

$$\begin{array}{c} v=[\begin{array}{c} v_{x}\\ v_{y}\\ v_{z} \end{array}]=[\begin{array}{c} \left(\text{cos}\;\phi (t)\;\text{sin}\;\theta (t)\;\text{cos}\;\psi (t)+\text{sin}\;\phi (t)\;\text{sin}\;\psi (t)\right)\frac{u_{1}}{m}\\ \left(\text{cos}\;\phi (t)\;\text{sin}\;\theta (t)\;\text{sin}\;\psi (t)-\text{sin}\;\phi (t)\;\text{cos}\;\psi (t)\right)\frac{u_{1}}{m}\\ (-g+\frac{1}{m}\left(\text{cos}\;\phi (t)\;\text{cos}\;\theta (t)\frac{u_{1}}{m}\right) \end{array}] \end{array}$$ (6)

According Eq (6), the total lift and tilting angles can be defined as follows:

$$\phi^{des}\left(t\right)=\text{arctan}(\text{cos}\;\theta^{des}\left(t\right)\frac{\text{sin}\;\psi^{des}\left(t\right)v_{x}-\text{cos}\;\psi^{des}\left(t\right)v_{y}}{v_{z}+g})$$ (7a)

$$\theta^{des}\left(t\right)=\text{arctan}(\frac{\text{cos}\;\psi^{des}\left(t\right)v_{x}+\text{sin}\;\psi^{des}\left(t\right)v_{y}}{v_{z}+g})$$ (7b)

$$u_{1}=m\sqrt{v_{x}^{2}+v_{y}^{2}+{\left(v_{z}+g\right)}^{2}}$$ (7c)

**Assumption 3**. *In this paper, the perturbation applied for each subsystem of the quadrotor, is bounded but unknown and satisfies* $|D_{i}\left(t\right)|\leq d_{i}$, *where d*_*i*_ > 0.

## 3 Finite-time control design for a quadrotor using a new hyper-plane based on integral non-singular SMC

**Fig 2:**
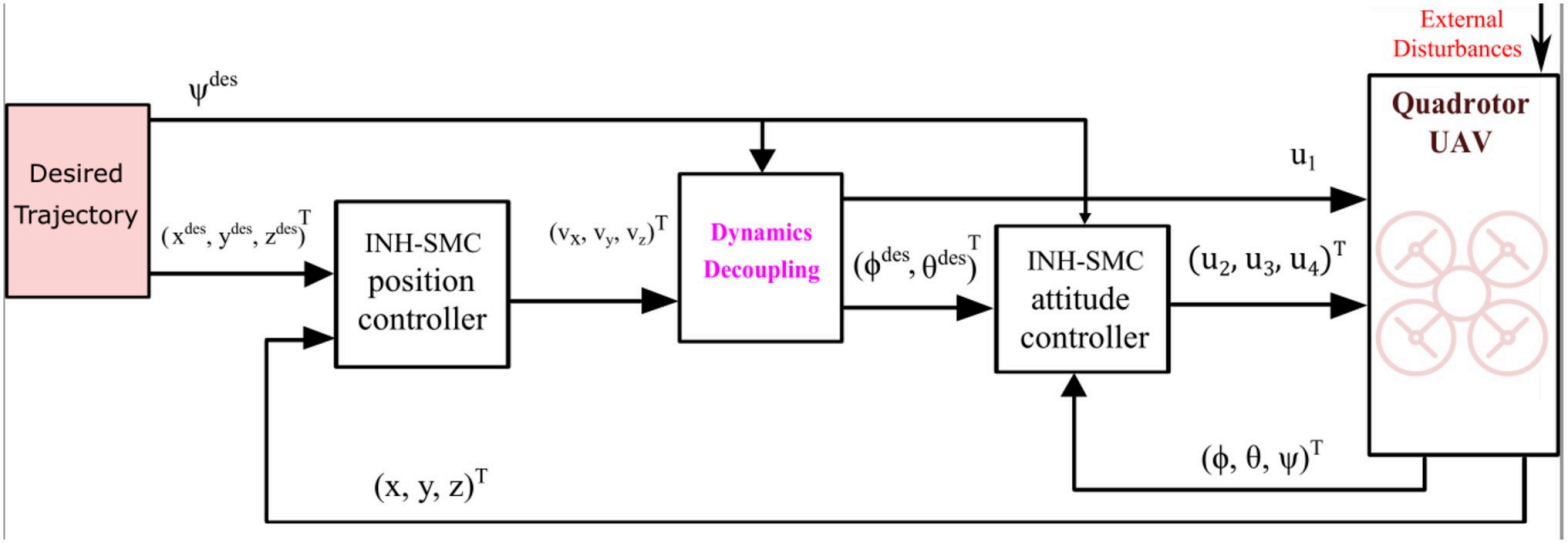
Structure block of the proposed control scheme.

In this section, a new control scheme is proposed for finite-time tracking control quadrotor system in the presence of external disturbances. Fig 2 shows the structure block of the proposed finite-time control for a quadrotor system. This finite-time control method is applied in the outer-loop control, which is realized by changing the attitude angles in the inner loop of a quadrotor. The outer-loop is used to generate the desired angles and the total thrust. The inner-loop is used to generate the rolling, pitching, and yawing torques. Two sliding mode variables are suggested for a quadrotor system, the first is an integral terminal sliding mode surface and the second is nonsingular TSMS. Based on these sliding manifolds, a new hyperplane-based sliding manifolds are developed for position/attitude subsystems. Then, the Lyapunov theory is used to prove the stability of the proposed controller.

### 3.1 New hyperplane-based sliding manifolds for a quadrotor position

A hyperplane-based sliding manifold is constructed using the integral terminal sliding mode (ITSM) [36] and the nonsingular terminal sliding mode (NTSM) [37] to achieve easy, precise, and robust tracking control for a quadrotor position.

Define the tracking errors of position as:

$$\begin{array}{c} e_{7}\left(t\right)=x\left(t\right)-x^{des}\left(t\right),\;e_{9}\left(t\right)=y\left(t\right)-y^{des}\left(t\right),\;e_{11}\left(t\right)=z\left(t\right)-z^{des}\left(t\right) \end{array}$$ (8)

The integral terminal sliding mode variable for the position can be described in order to ensure robustness and minimize steady state-errors as:

$$\begin{array}{c} \;\{\begin{array}{cc} \sigma_{7}\left(t\right) & =\Xi_{x1}e_{7}\left(t\right)+\Xi_{x2}\int {e_{7}\left(t\right)}^{\mu_{x}}sign\left(e_{7}\left(t\right)\right)dt\\ \sigma_{9}\left(t\right) & =\Xi_{y1}e_{9}\left(t\right)+\Xi_{y2}\int {e_{9}\left(t\right)}^{\mu_{y}}sign\left(e_{9}\left(t\right)\right)dt\\ \sigma_{11}\left(t\right) & =\Xi_{z1}e_{11}\left(t\right)+\Xi_{z2}\int {e_{11}\left(t\right)}^{\mu_{z}}sign\left(e_{11}\left(t\right)\right)dt \end{array} \end{array}$$ (9)

where Ξ_*i*1_ and Ξ_*i*2_ for *i* = *x*, *y*, *z* are positive parameters and $\frac{1}{2}<\mu_{i}<1$.

In order to achieve fast convergence and high tracking, hyperplane based sliding manifolds are designed using NTSM as follows:

$$\begin{array}{c} s_{7}\left(t\right)=\sigma_{7}\left(t\right)+\frac{1}{\beta_{x}}{\dot{\sigma }}_{7}^{\gamma_{x}}\left(t\right),\;\;s_{9}\left(t\right)=\sigma_{9}\left(t\right)+\frac{1}{\beta_{y}}{\dot{\sigma }}_{9}^{\gamma_{y}}\left(t\right),s_{11}\left(t\right)=\sigma_{11}\left(t\right)+\frac{1}{\beta_{z}}{\dot{\sigma }}_{11}^{\gamma_{z}}\left(t\right) \end{array}$$ (10)

in which, *β*_*x*,*y*,*z*_ is positive coefficient, and 1 < *γ*_*x*,*y*,*z*_ < 2. The designed hyperplane-based sliding manifold for position of a quadrotor is suggested to force *s*_7,9,11_(*t*) converge to zero for any conditions of *σ*_7,9,11_(*t*).

### 3.2 Finite-time control design for position loop

The controller introduced in this paper is composed of two control laws: one is a continuous control law, while the other is a discontinuous control law.

#### 3.2.1 Continuous control law for position loop

This component can be obtained by setting ${\dot{s}}_{7,9,11}\left(t\right)=0$ in without disturbances $D_{7,9,11}\left(t\right)=0$.

The time derivative of *σ*_*i*_ can be given by:

$$\begin{array}{cc} {\dot{s}}_{7,9,11}\left(t\right) & ={\dot{\sigma }}_{7,9,11}\left(t\right)+\frac{\gamma_{7,9,11}}{\beta_{7,9,11}}{\dot{\sigma }}_{7,9,11}^{\gamma_{7,9,11}-1}\left(t\right){\ddot{\sigma }}_{7,9,11}\left(t\right)\\ & =\frac{\gamma_{7,9,11}}{\beta_{7,9,11}}{\dot{\sigma }}_{7,9,11}^{\gamma_{7,9,11}-1}\left(t\right)\left(\frac{\beta_{7,9,11}}{\gamma_{7,9,11}}{\dot{\sigma }}_{i}^{2-\gamma_{7,9,11}}\left(t\right)+{\ddot{\sigma }}_{7,9,11}\left(t\right)\right) \end{array}$$ (11)

The time derivative of *σ*_7,9,11_(*t*) and its double time derivative are respectively given as:

$$\begin{array}{c} {\dot{\sigma }}_{7,9,11}\left(t\right)=\Xi_{i1}{\dot{e}}_{7,9,11}\left(t\right)+\Xi_{i2}|{e_{7,9,11}}^{|\mu_{i}}\left(t\right)sign\left(e_{7,9,11}\left(t\right)\right) \end{array}$$ (12)

and

$$\begin{array}{c} {\ddot{\sigma }}_{7,9,11}\left(t\right)=\Xi_{i1}{\ddot{e}}_{7,9,11}\left(t\right)+\Xi_{i2}\mu_{i}|e_{7,9,11}{|}^{\mu_{i}-1}\left(t\right){\dot{e}}_{7,9,11}\left(t\right) \end{array}$$ (13)

By assuming that (10) is equal to zero, one get:

$$\begin{array}{c} {\dot{e}}_{7,9,11}\left(t\right)=-\frac{\Xi_{i1}}{\Xi_{i2}}|e_{7,9,11}{|}^{\mu_{i}}\left(t\right)sign\left(e_{7,9,11}\left(t\right)\right) \end{array}$$ (14)

Submitting (14) in (12), the double time derivative of *s*_*i*_ is given by the following equation.

$$\begin{array}{c} {\ddot{\sigma }}_{7,9,11}\left(t\right)=\Xi_{i1}{\ddot{e}}_{7,9,11}\left(t\right)-\frac{\mu_{i}\Xi_{i2}^{2}}{\Xi_{i1}}|e_{7,9,11}{|}^{2\mu_{i}-1}\left(t\right)sign\left(e_{7,9,11}\left(t\right)\right) \end{array}$$ (15)

Submitting (15) in (11), it produces that

$$\begin{array}{c} {\dot{s}}_{7,9,11}\left(t\right)=\frac{\gamma_{i}}{\beta_{i}}{\dot{\sigma }}_{7,9,11}^{\gamma_{i}-1}\left(t\right)(\frac{\beta_{i}}{\gamma_{i}}{\dot{\sigma }}_{7,9,11}^{2-\gamma_{i}}\left(t\right)+\Xi_{i1}{\ddot{e}}_{7,9,11}\left(t\right)\\ -\frac{\mu_{i}\Xi_{i2}^{2}}{\Xi_{i1}}|e_{7,9,11}\left(t\right){|}^{2\mu_{i}-1}sign\left(e_{7,9,11}\left(t\right)\right)) \end{array}$$ (16)

By setting ${\dot{s}}_{7,9,11}\left(t\right)=0$ and tacking $D_{7,9,11}\left(t\right)=0$, the equivalent rule can be obtained. Then, using the double time derivative of the tracking errors, the continuous control laws for the position of a quadrotor are given by:

$$\begin{array}{cc} v_{xc} & =\frac{1}{\Xi_{x1}}(\frac{\mu_{x}\Xi_{x}^{2}}{\Xi_{x1}}|e_{7}\left(t\right){|}^{2\mu_{x}-1}sign\left(e_{7}\left(t\right)\right)-\frac{\beta_{x}}{\gamma_{x}}{\dot{s}}_{x}^{2-\gamma_{x}}-\Xi_{x1}M_{9}\dot{x}\left(t\right))\\ v_{yc} & =\frac{1}{\Xi_{y1}}(\frac{\mu_{y}\Xi_{y}^{2}}{\Xi_{y1}}|e_{9}\left(t\right){|}^{2\mu_{y}-1}sign\left(e_{9}\left(t\right)\right)-\frac{\beta_{y}}{\gamma_{y}}{\dot{s}}_{y}^{2-\gamma_{y}}-\Xi_{y1}M_{10}\dot{y}\left(t\right))\\ v_{zc} & =\frac{1}{\Xi_{z1}}(\frac{\mu_{z}\Xi_{z}^{2}}{\Xi_{z1}}|e_{11}\left(t\right){|}^{2\mu_{z}-1}sign\left(e_{11}\left(t\right)\right)-\frac{\beta_{z}}{\gamma_{z}}{\dot{s}}_{z}^{2-\gamma_{z}}-\Xi_{z1}M_{11}\dot{z}\left(t\right)+g) \end{array}$$ (17)

#### 3.2.2 Reaching control law for position loop

A switching law is added to the equivalent law to increase efficiency against model uncertainty/external disruption of a quadrotor device. Then its expressions can be given as follows:

$$\begin{array}{cc} v_{xs} & =\frac{1}{\Xi_{x1}}(-k_{x1}s_{7}\left(t\right)-k_{x2}sign\left(s_{7}\left(t\right)\right))\\ v_{ys} & =\frac{1}{\Xi_{y1}}(-k_{y1}s_{9}\left(t\right)-k_{y2}sign\left(s_{9}\left(t\right)\right))\\ v_{zs} & =\frac{1}{\Xi_{z1}}(-k_{z1}s_{11}\left(t\right)-k_{z2}sign\left(s_{11}\left(t\right)\right))\; \end{array}$$ (18)

where *k*_*i*1_ and *k*_*i*2_ for *i* = 7, 9, 11 are positive constants.

**Theorem 1**. *Consider the quadrotor position system* (5) *and the hyperplane-based sliding surfaces are designed in* (10) *and the control laws are designed in* (19), *then the tracking errors* (8) *of the closed-loop system can asymptotically converge to zero*.

$$\begin{array}{cc} v_{x} & =\frac{1}{\Xi_{x1}}(\begin{array}{c} \frac{\mu_{x}\Xi_{x}^{2}}{\Xi_{x1}}|e_{7}\left(t\right){|}^{2\mu_{x}-1}sign\left(e_{7}\left(t\right)\right)-\frac{\beta_{x}}{\gamma_{x}}{\dot{s}}_{x}^{2-\gamma_{x}}-\Xi_{x1}M_{9}\dot{x}\left(t\right)-k_{x1}s_{7}\left(t\right)\\ \;-k_{x2}sign\left(s_{7}\left(t\right)\right) \end{array})\\ v_{y} & =\frac{1}{\Xi_{y1}}(\begin{array}{c} \frac{\mu_{y}\Xi_{y}^{2}}{\Xi_{y1}}|e_{9}\left(t\right){|}^{2\mu_{y}-1}sign\left(e_{9}\left(t\right)\right)-\frac{\beta_{y}}{\gamma_{y}}{\dot{s}}_{y}^{2-\gamma_{y}}-\Xi_{y1}M_{10}\dot{y}\left(t\right)-k_{y1}s_{9}\left(t\right)\\ \;-k_{y2}sign\left(s_{9}\left(t\right)\right) \end{array})\\ v_{z} & =\frac{1}{\Xi_{z1}}(\begin{array}{c} \frac{\mu_{z}\Xi_{z}^{2}}{\Xi_{z1}}|e_{11}\left(t\right){|}^{2\mu_{z}-1}sign\left(e_{11}\left(t\right)\right)-\frac{\beta_{z}}{\gamma_{z}}{\dot{s}}_{z}^{2-\gamma_{z}}-\Xi_{z1}M_{11}\dot{z}\left(t\right)+g\\ \;-k_{z1}s_{11}\left(t\right)-k_{z2}sign\left(s_{11}\left(t\right)\right) \end{array}) \end{array}$$ (19)

*Proof*. Define a Lyapunov function for the position and attitude of a quadrotor in terms of *s*_7_(*t*), *s*_9_(*t*), and *s*_11_(*t*) as:

$$\begin{array}{c} V_{1}=0.5\left[s_{7}^{2}\left(t\right)+s_{9}^{2}\left(t\right)+s_{11}^{2}\left(t\right)\right] \end{array}$$ (20)

Differentiating *V*_Σ_, it yields

$$\begin{array}{c} {\dot{V}}_{1}=s_{7}\left(t\right){\dot{s}}_{7}\left(t\right)+s_{9}\left(t\right){\dot{s}}_{9}\left(t\right)+s_{11}\left(t\right){\dot{s}}_{11}\left(t\right) \end{array}$$ (21)

Now, by using (16) and (19),

$$\begin{array}{cc} {\dot{V}}_{1} & =s_{7}\left(t\right)\frac{\gamma_{x}}{\beta_{x}}{\dot{s}}_{7}^{\gamma_{x}-1}\left(t\right)\left[\Xi_{x1}D_{x}\left(t\right)-k_{x1}s_{7}\left(t\right)-k_{x2}sign\left(s_{7}\left(t\right)\right)\right]\\ \; & +s_{9}\left(t\right)\frac{\gamma_{y}}{\beta_{y}}{\dot{s}}_{9}^{\gamma_{y}-1}\left(t\right)\left[\Xi_{y1}D_{y}\left(t\right)-k_{y1}s_{9}\left(t\right)-k_{y2}sign\left(s_{9}\left(t\right)\right)\right]\\ \; & +s_{11}\left(t\right)\frac{\gamma_{z}}{\beta_{z}}{\dot{s}}_{11}^{\gamma_{z}-1}\left(t\right)\left[\Xi_{z1}D_{z}\left(t\right)-k_{z1}s_{11}\left(t\right)-k_{z2}sign\left(s_{11}\left(t\right)\right)\right] \end{array}$$ (22)

Using (11), the above equation leads to

$$\begin{array}{cc} {\dot{V}}_{1}\leq & -\frac{\gamma_{x}}{\beta_{x}}{\dot{s}}_{7}^{\gamma_{x}-1}\left(t\right)k_{x1}s_{7}^{2}\left(t\right)-\frac{\gamma_{y}}{\beta_{y}}{\dot{s}}_{9}^{\gamma_{y}-1}\left(t\right)k_{y1}s_{9}^{2}\left(t\right)-\frac{\gamma_{z}}{\beta_{z}}{\dot{s}}_{11}^{\gamma_{z}-1}\left(t\right)k_{z1}s_{11}^{2}\left(t\right)\\ & \;\leq 0 \end{array}$$ (23)

For any initial state *s*_7,9,11_(*t*) ≠ 0, define *t*_*ri*_ the reaching time to converge to zero. After that, *σ*_7,9,11_(*t*) will converge to zero as a consequence. The total time *t*_*fi*_ can be written as follows [37, 38]

$$\begin{array}{c} t_{fi}=t_{ri}+\frac{\gamma_{i}}{\gamma_{i}-1}\beta_{i}^{-\frac{1}{\gamma_{i}}}{\sigma (t_{ri})}^{\frac{\gamma_{i}-1}{\gamma_{i}}} \end{array}$$ (24)

As a result, the position tracking errors will asymptotically converge to zero.

The suggested controller is used for the position subsystem in this subsection, and the Lyapunov principle is used to prove the loop’s stability. In the next section, we’ll use the same steps we used for the position-loop to produce control torques, which stabilize the attitude-loop under disturbances.

### 3.3 Finite-time control design for attitude loop

By extracting the desired roll and pitch from the position control presented in the previous subsection, the torques of the quadrotor attitude can be designed in this section. Define the desired tracking errors of a quadrotor attitude as follows:

$$\begin{array}{c} e_{1}\left(t\right)=\phi \left(t\right)-\phi^{des}\left(t\right),\;e_{3}\left(t\right)=\theta \left(t\right)-\theta^{des}\left(t\right),\;e_{5}\left(t\right)=\psi \left(t\right)-\psi^{des}\left(t\right) \end{array}$$ (25)

The ITSM for the attitude can be described in order to ensure robustness and minimize steady state-errors as:

$$\begin{array}{c} \;\{\begin{array}{cc} \sigma_{1}\left(t\right) & =\Xi_{\phi 1}e_{1}\left(t\right)+\Xi_{\phi 2}\int |e_{1}\left(t\right){|}^{\mu_{\phi }}sign\left(e_{1}\left(t\right)\right)dt\\ \sigma_{3}\left(t\right) & =\Xi_{\theta 1}e_{3}\left(t\right)+\Xi_{\theta 2}\int |e_{3}\left(t\right){|}^{\mu_{\theta }}sign\left(e_{3}\left(t\right)\right)dt\\ \sigma_{5}\left(t\right) & =\Xi_{\psi 1}e_{5}\left(t\right)+\Xi_{\psi 2}\int |e_{5}\left(t\right){|}^{\mu_{\psi }}sign\left(e_{5}\left(t\right)\right)dt \end{array} \end{array}$$ (26)

where Ξ_*i*1_ and Ξ_*i*2_ for *i* = *ϕ*, *θ*, *ψ* are positive parameters and $\frac{1}{2}<\mu_{i}<1$.

In order to achieve fast convergence and high tracking, hyperplane based sliding manifolds are designed using NTSM as follows:

$$\begin{array}{c} s_{1}\left(t\right)=\sigma_{1}\left(t\right)+\frac{1}{\beta_{\phi }}{\dot{\sigma }}_{1}^{\gamma_{\phi }}\left(t\right),\;\;s_{3}\left(t\right)=\sigma_{3}\left(t\right)+\frac{1}{\beta_{\theta }}{\dot{\sigma }}_{3}^{\gamma_{\theta }}\left(t\right),s_{5}\left(t\right)=\sigma_{5}\left(t\right)+\frac{1}{\beta_{\psi }}{\dot{\sigma }}_{5}^{\gamma_{\psi }}\left(t\right) \end{array}$$ (27)

in which, *β*_*ϕ*,*θ*,*ψ*_ is positive coefficient, and 1 < *γ*_*ϕ*,*θ*,*ψ*_ < 2.

**Theorem 2**. *Consider the quadrotor attitude system* (5) *and the hyperplane-based sliding surfaces are designed in* (27) *and the control laws are designed in* (28), *then the tracking errors* (25) *of the closed-loop system can asymptotically converge to zero*.

$$\begin{array}{cc} u_{2} & =\frac{1}{N_{1}\Xi_{\phi 1}}(\begin{array}{c} \frac{\mu_{\phi }\Xi_{\phi }^{2}}{\Xi_{\phi 1}}|e_{1}\left(t\right){|}^{2\mu_{\phi }-1}sign\left(e_{1}\left(t\right)\right)-\frac{\beta_{\phi }}{\gamma_{\phi }}{\dot{\sigma }}_{1}^{2-\gamma_{\phi }}\left(t\right)-\Xi_{\phi 1}\{M_{1}\dot{\theta }\left(t\right)\dot{\psi }\left(t\right)\\ \;+M_{2}\dot{\theta }\left(t\right)+M_{3}{\dot{\phi }}^{2}\left(t\right)]+{\ddot{\phi }}^{des}\left(t\right)-k_{\phi 1}s_{1}\left(t\right)-k_{\phi 2}sign\left(s_{1}\left(t\right)\right\} \end{array})\\ u_{3} & =\frac{1}{N_{2}\Xi_{\theta 1}}(\begin{array}{c} \frac{\mu_{\theta }\Xi_{\theta }^{2}}{\Xi_{\theta 1}}|e_{3}\left(t\right){|}^{2\mu_{\theta }-1}sign\left(e_{3}\left(t\right)\right)-\frac{\beta_{\theta }}{\gamma_{\theta }}{\dot{\sigma }}_{3}^{2-\gamma_{\theta }}\left(t\right)+\Xi_{\theta 1}\{M_{4}\dot{\phi }\left(t\right)\dot{\psi }\left(t\right)\\ \;+M_{5}\dot{\phi }\left(t\right)+M_{6}{\dot{\theta }}^{2}\left(t\right)+{\ddot{\theta }}^{des}\left(t\right)-k_{\theta 1}s_{3}\left(t\right)-k_{\theta 2}sign\left(s_{3}\left(t\right)\right)\} \end{array})\\ u_{4} & =\frac{1}{N_{3}\Xi_{\psi 1}}(\begin{array}{c} \frac{\mu_{\psi }\Xi_{\psi }^{2}}{\Xi_{\psi 1}}|e_{5}\left(t\right){|}^{2\mu_{\psi }-1}sign\left(e_{5}\left(t\right)\right)-\frac{\beta_{\psi }}{\gamma_{\psi }}{\dot{\sigma }}_{5}^{2-\gamma_{\psi }}\left(t\right)+\Xi_{\psi 1}\{M_{7}\dot{\phi }\left(t\right)\dot{\theta }\left(t\right)\\ \;+M_{8}{\dot{\psi }}^{2}\left(t\right)+{\ddot{\psi }}^{des}\left(t\right)-k_{\psi 1}s_{5}\left(t\right)-k_{\psi 2}sign\left(s_{5}\left(t\right)\right)\} \end{array}) \end{array}$$ (28)

*Proof*. Define a Lyapunov function for the position and attitude of a quadrotor in terms of *s*_1_(*t*), *s*_3_(*t*), and *s*_5_(*t*) as:

$$\begin{array}{c} V_{2}=0.5\left[s_{1}^{2}\left(t\right)+s_{3}^{2}\left(t\right)+s_{5}^{2}\left(t\right)\right] \end{array}$$ (29)

Differentiating *V*_2_, it yields

$$\begin{array}{c} {\dot{V}}_{2}=s_{1}\left(t\right){\dot{s}}_{1}\left(t\right)+s_{3}\left(t\right){\dot{s}}_{3}\left(t\right)+s_{5}\left(t\right){\dot{s}}_{5}\left(t\right) \end{array}$$ (30)

Now, by using the time derivative of sliding mode variables and (28),

$$\begin{array}{cc} {\dot{V}}_{1}= & s_{1}\left(t\right)\frac{\gamma_{\phi }}{\beta_{\phi }}{\dot{s}}_{1}^{\gamma_{\phi }-1}\left(t\right)\left[\Xi_{\phi 1}D_{\phi }\left(t\right)-k_{\phi 1}s_{1}\left(t\right)-k_{\phi 2}sign\left(s_{1}\left(t\right)\right)\right]\\ \; & +s_{3}\left(t\right)\frac{\gamma_{\theta }}{\beta_{\theta }}{\dot{s}}_{3}^{\gamma_{\theta }-1}\left(t\right)\left[\Xi_{\theta 1}D_{\theta }\left(t\right)-k_{\theta 1}s_{3}\left(t\right)-k_{\theta 2}sign\left(s_{3}\left(t\right)\right)\right]\\ \; & +s_{5}\left(t\right)\frac{\gamma_{\psi }}{\beta_{\psi }}{\dot{s}}_{5}^{\gamma_{\psi }-1}\left(t\right)\left[\Xi_{\psi 1}D_{\psi }\left(t\right)-k_{\psi 1}s_{5}\left(t\right)-k_{\psi 2}sign\left(s_{5}\left(t\right)\right)\right] \end{array}$$ (31)

Using the time derivative of sliding mode variables of attitude loop, the above equation leads to

$$\begin{array}{cc} {\dot{V}}_{1}\leq & -\frac{\gamma_{\phi }}{\beta_{\phi }}{\dot{s}}_{1}^{\gamma_{\phi }-1}\left(t\right)k_{\phi 1}s_{1}^{2}\left(t\right)-\frac{\gamma_{\theta }}{\beta_{\theta }}{\dot{s}}_{3}^{\gamma_{\theta }-1}\left(t\right)k_{\theta 1}s_{3}^{2}\left(t\right)-\frac{\gamma_{\psi }}{\beta_{\psi }}{\dot{s}}_{5}^{\gamma_{\psi }-1}\left(t\right)k_{\psi 1}s_{5}^{2}\left(t\right)\\ & \;\leq 0 \end{array}$$ (32)

For any initial state *s*_1,3,5_(*t*) ≠ 0, define *t*_*rj*_ the reaching time to converge to zero. After that, *σ*_1,3,5_(*t*) will converge to zero as a consequence. The total time *t*_*fj*_ can be written as follows [37, 38]

$$\begin{array}{c} t_{fj}=t_{rj}+\frac{\gamma_{j}}{\gamma_{j}-1}\beta_{j}^{-\frac{1}{\gamma_{j}}}{\sigma (t_{rj})}^{\frac{\gamma_{j}-1}{\gamma_{j}}} \end{array}$$ (33)

As a result, the position tracking errors will asymptotically converge to zero.

### 3.4 Stability analysis of closed loop system

The following Theorem shows the results of the proposed controller and the stability of the closed-loop system is provided.

**Theorem 3**. *Consider the quadrotor system* (5) *and the hyperplane-based sliding surfaces s are designed as* (10), (27) *and the control laws are designed as* (19) and (28), *then the tracking errors* (8) *and* (25) *of the closed-loop system can asymptotically converge to zero*.

*Proof*. Define a Lyapunov function for the position and attitude of a quadrotor in terms of *s*_7_(*t*), *s*_9_(*t*), *s*_11_(*t*), *s*_1_(*t*), *s*_3_(*t*), and *s*_5_(*t*) as:

$$\begin{array}{c} V_{12}=0.5\left[s_{7}^{2}\left(t\right)+s_{9}^{2}\left(t\right)+s_{11}^{2}\left(t\right)+s_{1}^{2}\left(t\right)+s_{3}^{2}\left(t\right)+s_{5}^{2}\left(t\right)\right] \end{array}$$ (34)

Differentiating *V*_12_, it yields

$$\begin{array}{c} {\dot{V}}_{12}=s_{7}\left(t\right){\dot{s}}_{7}\left(t\right)+s_{9}\left(t\right){\dot{s}}_{9}\left(t\right)+s_{11}\left(t\right){\dot{s}}_{11}\left(t\right)+s_{1}\left(t\right){\dot{s}}_{1}\left(t\right)+s_{3}\left(t\right){\dot{s}}_{3}\left(t\right)+s_{5}\left(t\right){\dot{s}}_{5}\left(t\right) \end{array}$$ (35)

Now, by using the time derivative of position and attitude sliding mode variables, (19), and (28),

$$\begin{array}{cc} {\dot{V}}_{12}= & s_{7}\left(t\right)\frac{\gamma_{x}}{\beta_{x}}{\dot{s}}_{x}^{\gamma_{x}-1}\left[\Xi_{x1}D_{x}\left(t\right)-k_{x1}s_{7}\left(t\right)-k_{x2}sign\left(s_{7}\left(t\right)\right)\right]\\ \; & +s_{9}\left(t\right)\frac{\gamma_{y}}{\beta_{y}}{\dot{s}}_{y}^{\gamma_{y}-1}\left[\Xi_{y1}D_{y}\left(t\right)-k_{y1}s_{9}\left(t\right)-k_{y2}sign\left(s_{9}\left(t\right)\right)\right]\\ \; & +s_{11}\left(t\right)\frac{\gamma_{z}}{\beta_{z}}{\dot{s}}_{z}^{\gamma_{z}-1}\left[\Xi_{z1}D_{z}\left(t\right)-k_{z1}s_{11}\left(t\right)-k_{z2}sign\left(s_{11}\left(t\right)\right)\right]\\ \; & +s_{1}\left(t\right)\frac{\gamma_{\phi }}{\beta_{\phi }}{\dot{s}}_{\phi }^{\gamma_{\phi }-1}\left[\Xi_{\phi 1}D_{\phi }-k_{\phi 1}s_{1}\left(t\right)-k_{\phi 2}sign\left(s_{1}\left(t\right)\right)\right]\\ \; & +s_{3}\left(t\right)\frac{\gamma_{\theta }}{\beta_{\theta }}{\dot{s}}_{\theta }^{\gamma_{\theta }-1}\left[\Xi_{\theta 1}D_{\theta }-k_{\theta 1}s_{3}\left(t\right)-k_{\theta 2}sign\left(s_{3}\left(t\right)\right)\right]\\ \; & +s_{5}\left(t\right)\frac{\gamma_{\psi }}{\beta_{\psi }}{\dot{s}}_{\psi }^{\gamma_{\psi }-1}\left[\Xi_{\psi 1}D_{\psi }-k_{\psi 1}s_{5}\left(t\right)-k_{\psi 2}sign\left(s_{5}\left(t\right)\right)\right] \end{array}$$ (36)

Using (23) and (32), the above equation leads to

$$\begin{array}{cc} {\dot{V}}_{12}\leq & \frac{\gamma_{x}}{\beta_{x}}{\dot{s}}_{x}^{\gamma_{x}-1}\left[\right|s_{7}\left(t\right)|(\Xi_{x1}|D_{x}\left(t\right)|-k_{x2})-k_{x1}s_{7}^{2}\left(t\right)]\\ \; & +\frac{\gamma_{y}}{\beta_{y}}{\dot{s}}_{y}^{\gamma_{y}-1}\left[\right|s_{9}\left(t\right)|(\Xi_{y1}|D_{y}\left(t\right)|-k_{y2})-k_{y1}s_{9}^{2}\left(t\right)]\\ \; & +\frac{\gamma_{z}}{\beta_{z}}{\dot{s}}_{z}^{\gamma_{z}-1}\left[\right|s_{11}\left(t\right)|(\Xi_{z1}|D_{z}\left(t\right)|-k_{z2})-k_{z1}s_{11}^{2}\left(t\right)]\\ \; & +\frac{\gamma_{\phi }}{\beta_{\phi }}{\dot{s}}_{\phi }^{\gamma_{\phi }-1}\left[\right|s_{1}\left(t\right)|(\Xi_{\phi 1}|D_{\phi }|-k_{\phi 2})-k_{\phi 1}s_{1}^{2}\left(t\right)]\\ \; & +\frac{\gamma_{\theta }}{\beta_{\theta }}{\dot{s}}_{\theta }^{\gamma_{\theta }-1}\left[\right|s_{3}\left(t\right)|(\Xi_{\theta 1}|D_{\theta }|-k_{\theta 2})-k_{\theta 1}s_{3}^{2}\left(t\right)]\\ \; & +\frac{\gamma_{\psi }}{\beta_{\psi }}{\dot{s}}_{\psi }^{\gamma_{\psi }-1}\left[\right|s_{5}\left(t\right)|(\Xi_{\psi 1}|D_{\psi }|-k_{\psi 2})-k_{\psi 1}s_{5}^{2}\left(t\right)] \end{array}$$ (37)

We choose $k_{x2}>\Xi_{x1}\left|D_{x}\left(t\right)\right|$, $k_{y2}>\Xi_{y1}\left|D_{y}\left(t\right)\right|$, $k_{z2}>\Xi_{z1}\left|D_{z}\left(t\right)\right|$, $k_{\phi 2}>\Xi_{\phi 1}\left|D_{\phi }\right|$, $k_{\theta 2}>\Xi_{\theta 1}\left|D_{\theta }\right|$, and $k_{\psi 2}>\Xi_{\psi 1}\left|D_{\psi }\right|$. Eq (37) becomes:

$$\begin{array}{cc} {\dot{V}}_{12}\leq & -\frac{\gamma_{x}}{\beta_{x}}{\dot{s}}_{x}^{\gamma_{x}-1}k_{x1}s_{7}^{2}\left(t\right)-\frac{\gamma_{y}}{\beta_{y}}{\dot{s}}_{x}^{\gamma_{y}-1}k_{y1}s_{9}^{2}\left(t\right)-\frac{\gamma_{z}}{\beta_{z}}{\dot{s}}_{z}^{\gamma_{z}-1}k_{z1}s_{11}^{2}\left(t\right)\\ & \;-\frac{\gamma_{\phi }}{\beta_{\phi }}{\dot{s}}_{\phi }^{\gamma_{\phi }-1}k_{\phi 1}s_{1}^{2}\left(t\right)-\frac{\gamma_{\theta }}{\beta_{\theta }}{\dot{s}}_{\theta }^{\gamma_{\theta }-1}k_{\theta 1}s_{3}^{2}\left(t\right)-\frac{\gamma_{\psi }}{\beta_{\psi }}{\dot{s}}_{\psi }^{\gamma_{\psi }-1}k_{\psi 1}s_{5}^{2}\left(t\right)\\ & \;\leq 0 \end{array}$$ (38)

Hence, the tracking errors of the position and attitude can asymptotically converge to zero. The proof is thus completed.

## 4 Simulation results

Numerical simulations are used to evaluate the tracking performance of the hyperplane-based sliding mode controller described in this paper. As a comparison, the super-twisting PID sliding mode controller provided in [17] is used.

### 4.1 Control parameters selection

During the simulation, the user has to select the value that achieves the best balance of tracking accuracy and control smoothness. The discussion of the controller parameter selection for the proposed control technique is summarized as follows:

- Selections of Ξ_*i*1_, Ξ_*i*2_, and *μ*_*i*_ for *i* = *x*, *y*, *z*, *ϕ*, *θ*, *ψ*: The parameters Ξ_*i*1_, Ξ_*i*2_, and *μ*_*i*_ are used in ITSM manifolds as given in (9) and (26). Faster convergence of the tracking errors can be obtained by choosing a smaller value of Ξ_*i*1_Ξ_*i*2_, and $\frac{1}{2}<\mu_{i}<1$ For a quadrotor position and attitude loop, Ξ_*i*1_ = 1, Ξ_*i*2_ = 0.0046, and *μ*_*i*_ = 1 are selected.
- Selections of *β*_*i*_ and *γ*_*i*_ for *i* = *x*, *y*, *z*, *ϕ*, *θ*, *ψ*: The gains *β*_*i*_ and *γ*_*i*_ are used in NTSM as shown in (10) for quadrotor position and (27) for altitude loop. Faster convergence of the tracking errors can be obtained by choosing a high positive value of *β*_*i*_, and a smaller value of 1 < *γ*_*i*_ < 2, however, it increases the magnitude of the control effort. The best choice of NTSM gains is presented in Table 2
- Selections of *k*_*i*1_ and *k*_*i*2_ for *i* = *x*, *y*, *z*, *ϕ*, *θ*, *ψ*: The positive gains *k*_*i*1_ and *k*_*i*2_ are used in the switching law (18) affect the robustness of the system by balancing the control signal smoothness. The selective values of *k*_*i*1_ and *k*_*i*2_ parameters are presented in Table 2.

**Remark 1**. *The design parameters of the controllers need to be tuned to achieve the satisfactory performance in terms of quadrotor trajectory-tracking in the presence of disturbances. To pick the optimal values for such parameters, the optimization toolbox in MATLAB program has been used (see Ref.* [39]).

Tables 1 and 2 list the controller and quadrotor parameters, respectively. The following are the beginning conditions for quadrotor states:

$$\begin{array}{cc} x_{0} & =0.05m,\;y_{0}=1m,\;z_{0}=0.01m,\\ \phi_{0} & =0rad,\;\theta_{0}=0rad,\;\psi_{0}=0rad \end{array}$$ (39)

Two scenarios in terms of disturbances path following are proposed in this section.

**Table 1:** Quadrotor parameters.

Parameter	Value	Parameter	Value
*g*(*s*−^2^.*m*)	9.8	*K*_5_(*Nms*^2^)	0.01
*m*(*kg*)	0.486	*K*_6_(*Nms*^2^)	0.01
*I*_*x*_(*m*−^2^.*kg*)	3.8278e-3	*K*_1_(*Nrads*^2^)	0.012
*I*_*y*_(*m*−^2^.*kg*)	3.8278e-3	*K*_2_(*Nrads*^2^)	0.012
*I*_*z*_(*m*−^2^.*kg*)	7.6566e-3	*K*_3_(*Nrads*^2^)	0.012
*J*_*r*_(*m*−^2^.*kg*)	2.8385e-5	*ρ*(*N*.*s*^2^)	2.9842e-3
*K*_4_(*Nms*^2^)	0.01	*ρ*_*c*_(*N*.*m*.*s*^2^)	3.2320e-2

**Table 2:** Control system parameters.

Parameter	Value	Parameter	Value
*μ*_*ϕ*,*θ*,*ψ*_	1	*β*_*ϕ*,*θ*,*ψ*_	102.15
*γ*_*ϕ*,*θ*,*ψ*_	1.9	Ξ_*i*2_	0.0046
Ξ_*i*1_	1	*k*_*ϕ*2,*θ*2,*ψ*2_	2.6997
*k*_*ϕ*1,*θ*1,*ψ*1_	817.6194	*k*_*x*1,*y*1,*z*1_	6
*μ*_*x*,*y*,*z*_	1	*β*_*x*,*y*,*z*_	2.1487
*γ*_*x*,*y*,*z*_	1.1	*k*_*x*2,*y*2,*z*2_	2

### 4.2 Simulation 1

**Fig 3:**
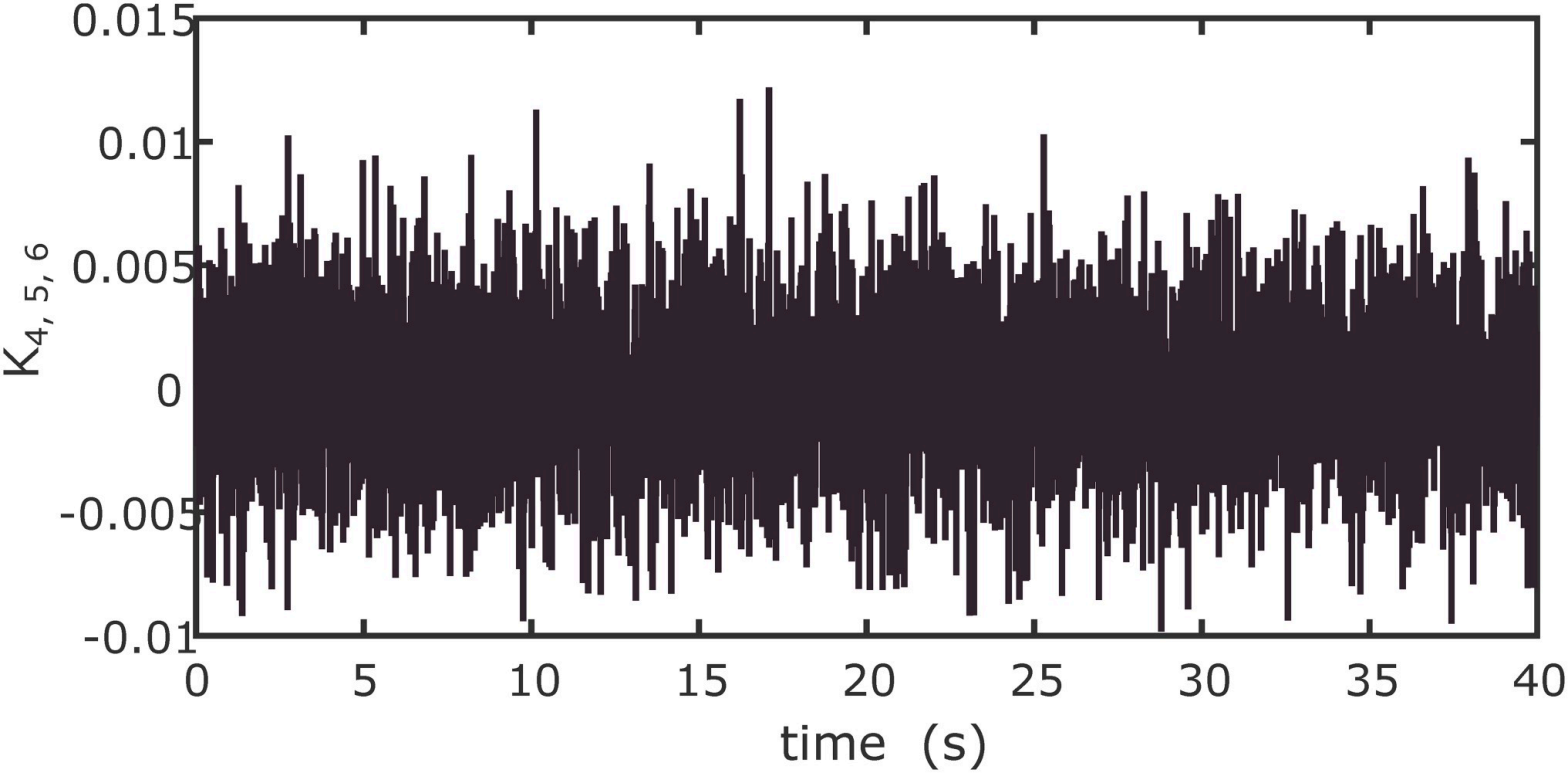
Drag coefficients for translational motion.

**Fig 4:**
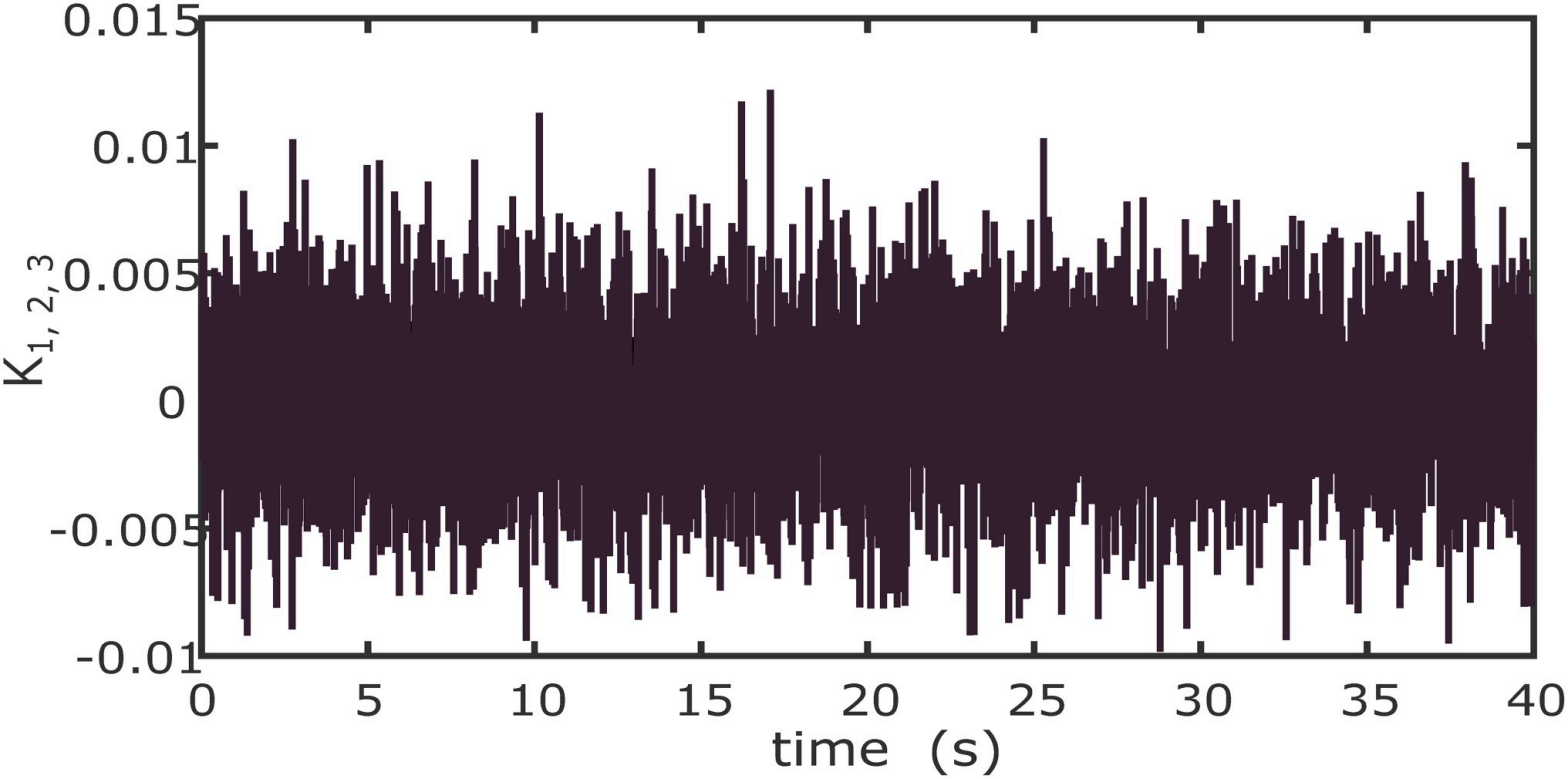
Drag coefficients for rotational motion.

In order to examine the tracking performance of the proposed control scheme, the complex change of the drag coefficients is considered in this scenario. This effect is shown in Figs 3 and 4, respectively for translational and rotational subsystems. The desired path used in this simulation is given by:

$$\begin{array}{cc} & x^{des}\left(t\right)=\{\begin{array}{c} 0.5\;\text{cos}(0.5t)\;m\\ 0.5\;m\\ 0.25t-4.5\;m\\ 3\;m \end{array}\begin{array}{c} t\in [0,4\pi )\\ t\in [4\pi ,20)\\ t\in [20,30)\\ t\in [30,80] \end{array}y^{des}\left(t\right)=\{\begin{array}{c} 0.5\;\text{sin}(0.5t)\;m\\ 0.25t-3.14\;m\\ 5-\pi \;m\\ -0.2358t+8.94\;m\\ -0.5\;m \end{array}\begin{array}{c} t\in [0,4\pi )\\ t\in [4\pi ,20)\\ t\in [20,30)\\ t\in [30,40]\\ t\in [40,80] \end{array} \end{array}$$ (40)

$$\begin{array}{cc} & z^{des}\left(t\right)=\{\begin{array}{c} 0.125t+1\;m\\ 0.5\pi +1\;m\\ \text{exp}(-0.2t+8.944)\;m \end{array}\begin{array}{c} t\in [0,4\pi )\\ t\in [4\pi ,40)\\ t\in [40,80) \end{array}\psi^{des}\left(t\right)=\{\begin{array}{c} \frac{\pi }{4}\;rad\\ 0\;rad \end{array}\begin{array}{c} t\in [0,50)\;\\ t\in (50,80] \end{array} \end{array}$$ (41)

$$\begin{array}{cc} x^{des}\left(t\right) & =\text{sin}\left(0.5t\right)m,\;y^{des}\left(t\right)=\text{cos}\left(0.5t\right)m,\;z^{des}\left(t\right)=0.1t+2m,\\ & \;\psi^{des}\left(t\right)=0.3rad \end{array}$$ (42)

External disturbances used in this simulation for quadrotor position and attitude are set as follows:

$$\begin{array}{cc} D_{x}\left(t\right) & =1+\text{sin}\left(0.2\pi t\right)\;m/s^{2}\\ D_{y}\left(t\right) & =1+cos\left(0.2\pi t\right)\;m/s^{2}\\ D_{z}\left(t\right) & =0.5\;\text{cos}\left(0.7t\right)+0.7\;\text{sin}\left(0.3\right)\;m/s^{2}\\ D_{\phi }\left(t\right) & =2\;\text{sin}\left(0.7t\right)+1\;rad/s^{2}\\ D_{\theta }\left(t\right) & =2\;\text{cos}\left(0.9t\right)+1\;rad/s^{2}\\ D_{\psi }\left(t\right) & =2\;\text{tanh}\left(0.7t\right)\;rad/s^{2} \end{array}$$ (43)

**Fig 5:**
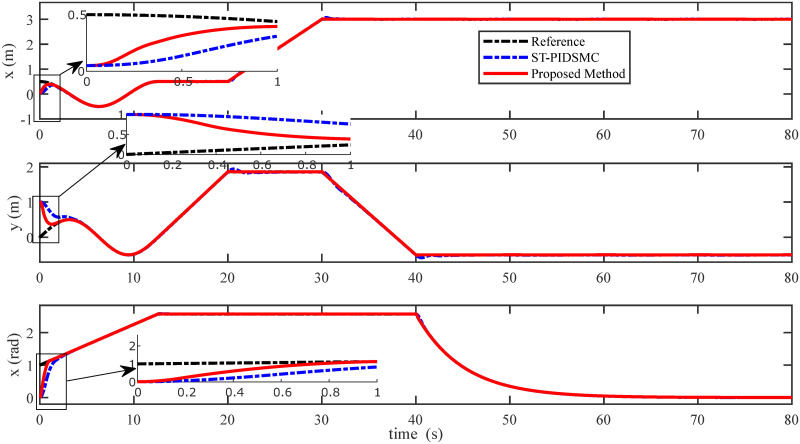
Results of the position under the proposed and ST-PID-SM controllers.

**Fig 6:**
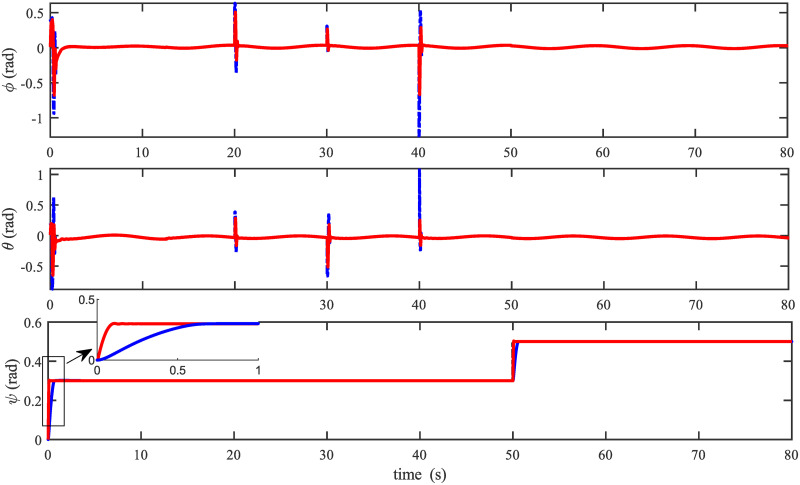
Results of the attitude under the proposed and ST-PID-SM controllers.

**Fig 7:**
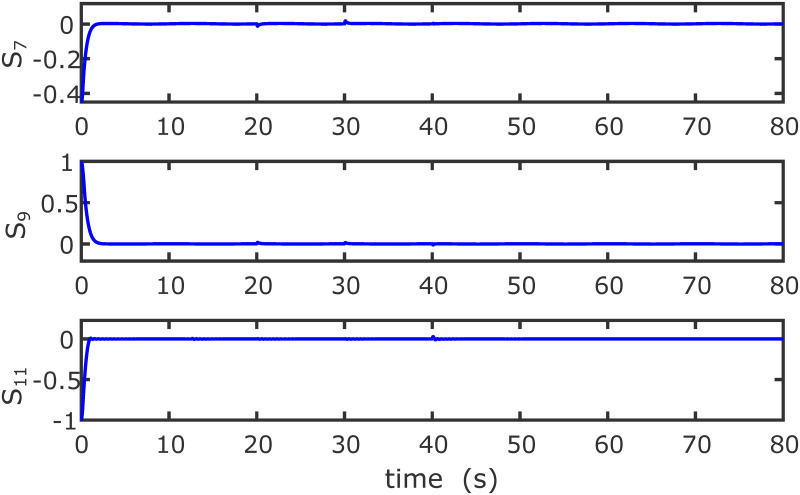
Results of the position sliding variables under the proposed controller.

**Fig 8:**
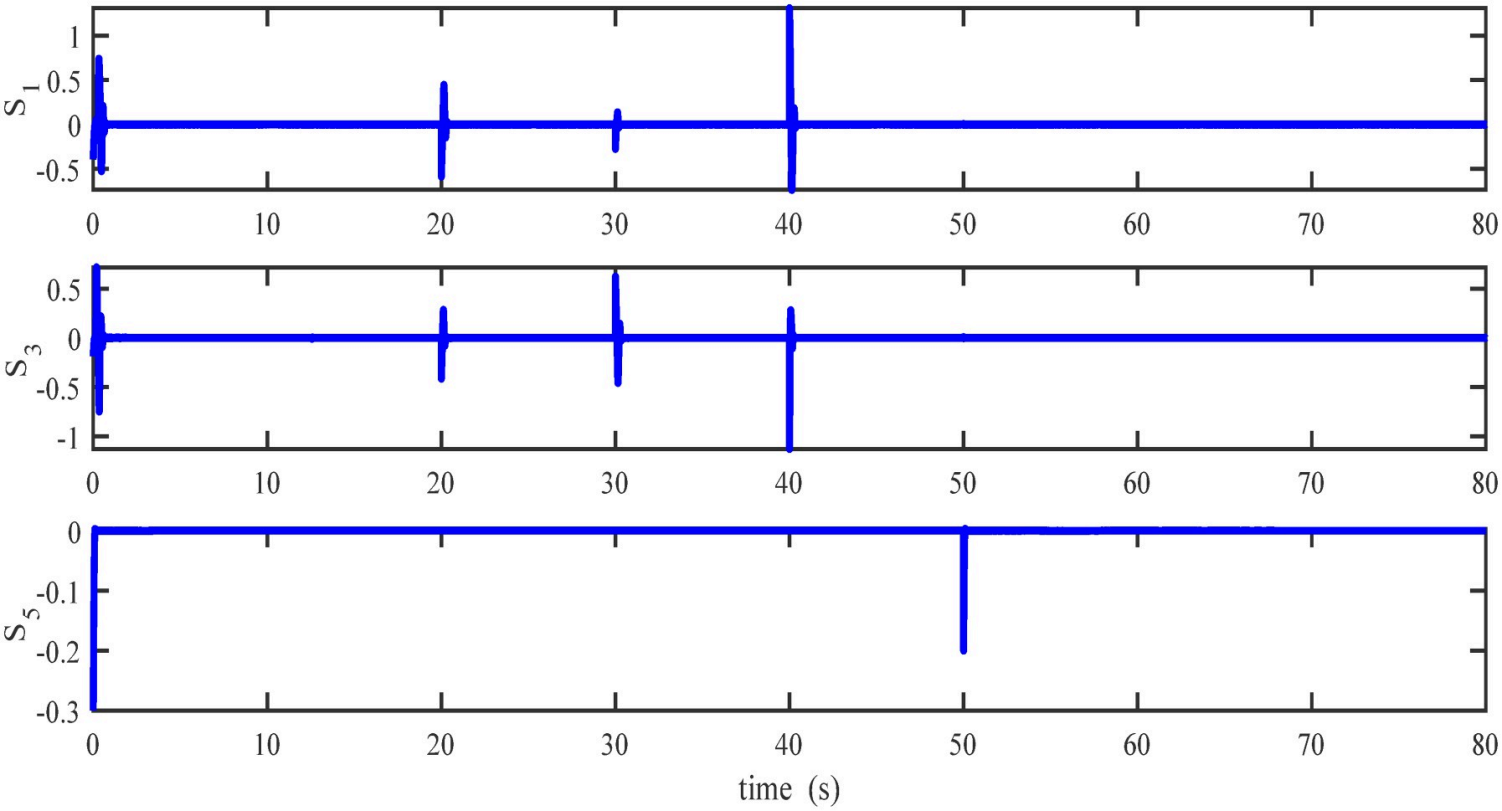
Results of the attitude sliding variables under the proposed controller.

**Fig 9:**
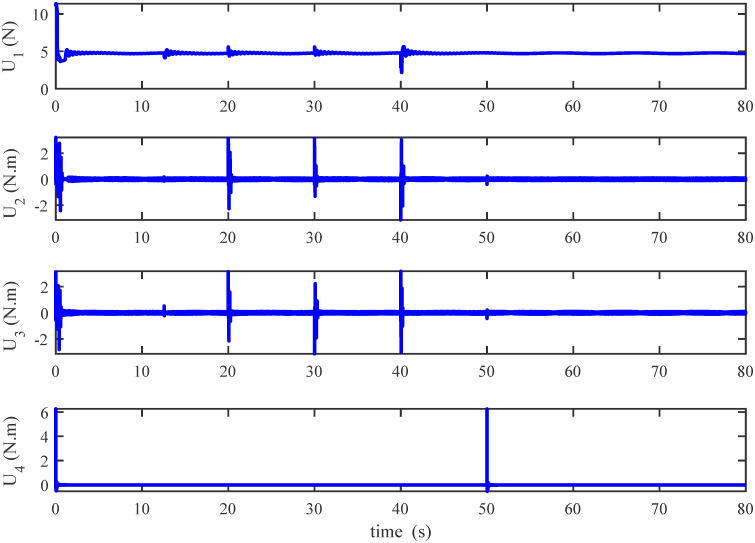
Results of the inputs under the proposed controller.

**Fig 10:**
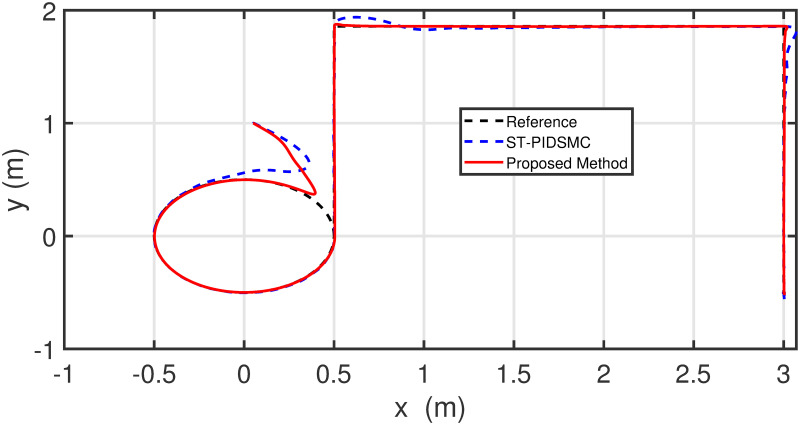
Results of the path following in 2D space under the proposed controller.

**Fig 11:**
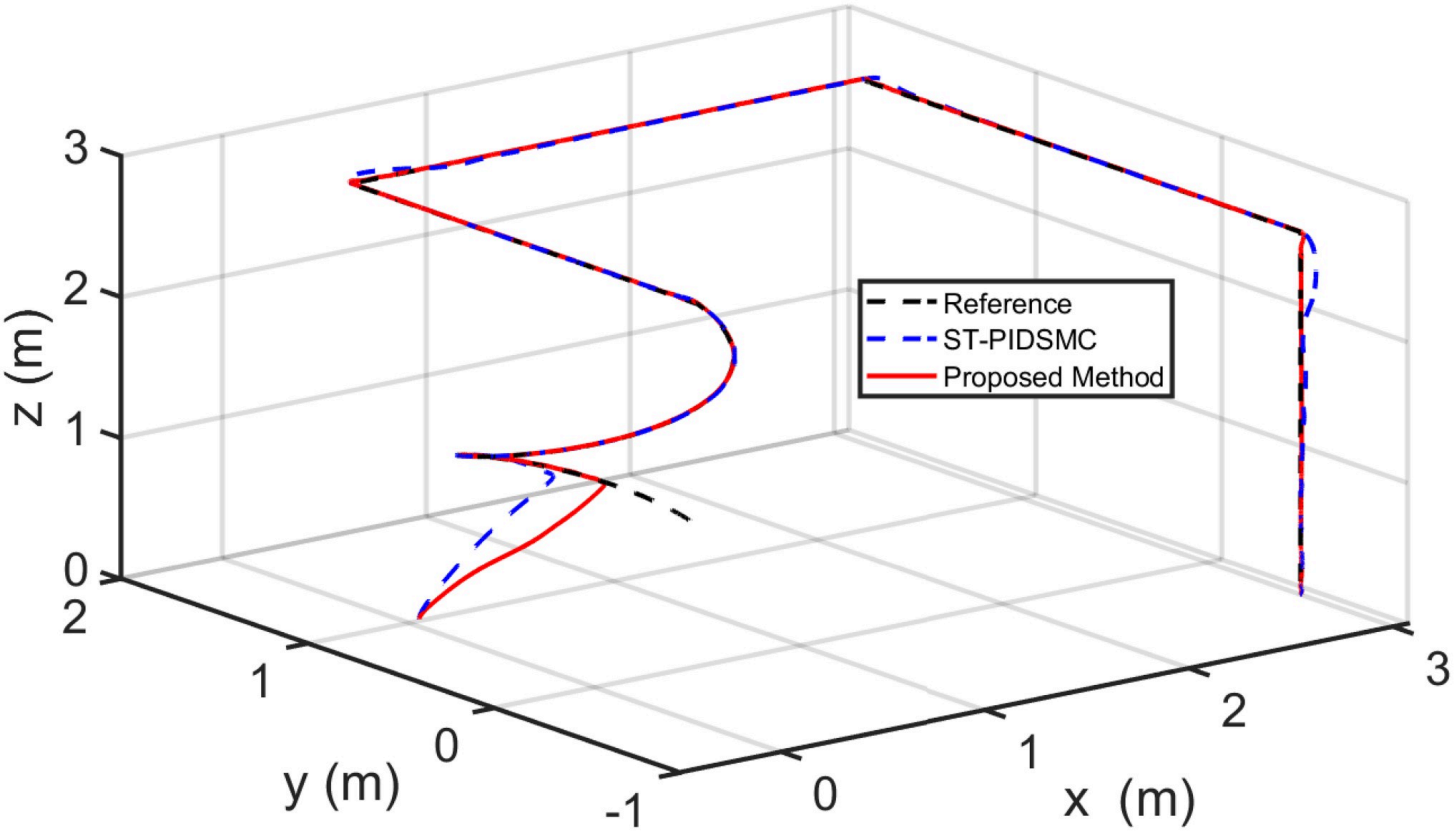
Results of the path following in 3D space under the proposed controller.

Figs 5 to 11 demonstrate the trajectory tracking responses utilizing the control methodology suggested in this paper and a super-twisting PID sliding mode controller. The absolute position result is plotted in Fig 5; as can be seen from these results, the proposed controller ensures that the quadrotor follows the desired trajectory with great precision, even when external disturbances are present. The suggested controller produced faster position responses than the SP-PIDSMC approach, as illustrated in Fig 5. The roll, pitch, and yaw angles converge to their intended angles in a short finite time, as shown in Fig 6. The vehicle is more stable under the proposed controller under the disturbed flight. Figs 7 and 8 depict the time trajectories of the sliding surfaces of the quadrotor’s position and attitude, which converge in finite time to their target trajectories. The total thrust and control torques (e.g. rolling, pitching, and yawing torques) are shown in Fig 9, demonstrating the chattering free of replies. The signal inputs provided by the proposed controller are smooth and have appropriate amplitudes, as shown in Fig 9. The quadrotor follows the intended trajectory, as shown in Fig 11. Fig 10 shows trajectory of output on *xOy* plane. Finally, the results show that, when compared to the ST-PID sliding mode controller, the suggested control method ensures more the quadrotor’s stability when subjected to external disturbances.

### 4.3 Simulation 2

Additional scenario called simulation 2 were carried out to verify the proposed controller’s robustness against disturbances. To simulate the external disturbances, we inject a time-varying disturbance into the model of a quadrotor, as presented in the following equations.

$$\begin{array}{cc} D_{x}\left(t\right) & =0.5\;\text{sin}\left(t\right)\;m/s^{2}\\ D_{y}\left(t\right) & =0.1\;\text{cos}\left(t\right)\;m/s^{2}\\ D_{z}\left(t\right) & =0.5\;\text{sin}\left(t\right)\;\text{cos}\left(t\right)\;m/s^{2}\\ D_{\phi }\left(t\right) & =0.5\;\text{cos}\left(0.5t\right)\;rad/s^{2}\\ D_{\theta }\left(t\right) & =0.5\;\text{sin}\left(0.5t\right)\;rad/s^{2}\\ D_{\psi }\left(t\right) & =0.5\;\text{sin}\left(0.7t\right)\;\text{cos}\left(0.7t\right)\;rad/s^{2} \end{array}$$ (44)

**Fig 12:**
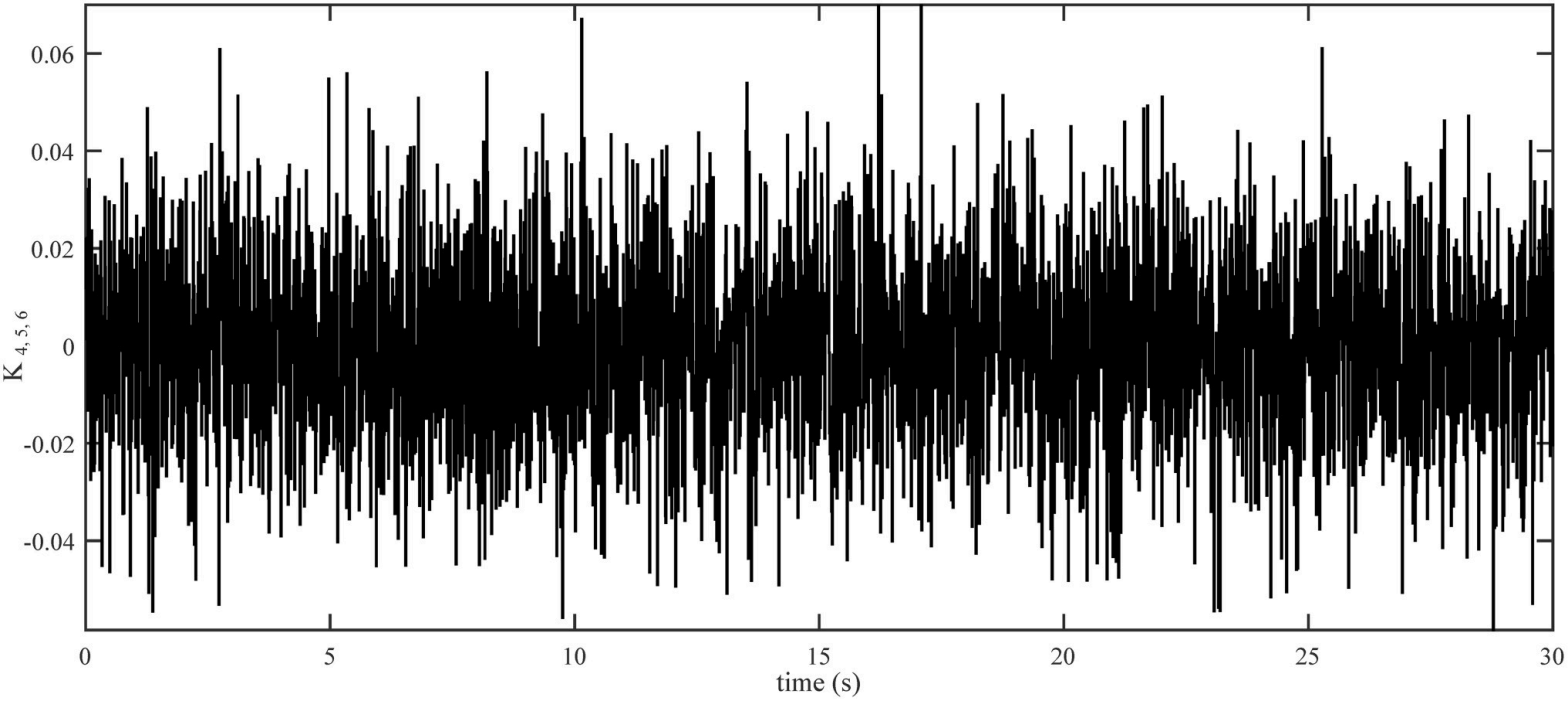
Drag coefficients for translational motion.

**Fig 13:**
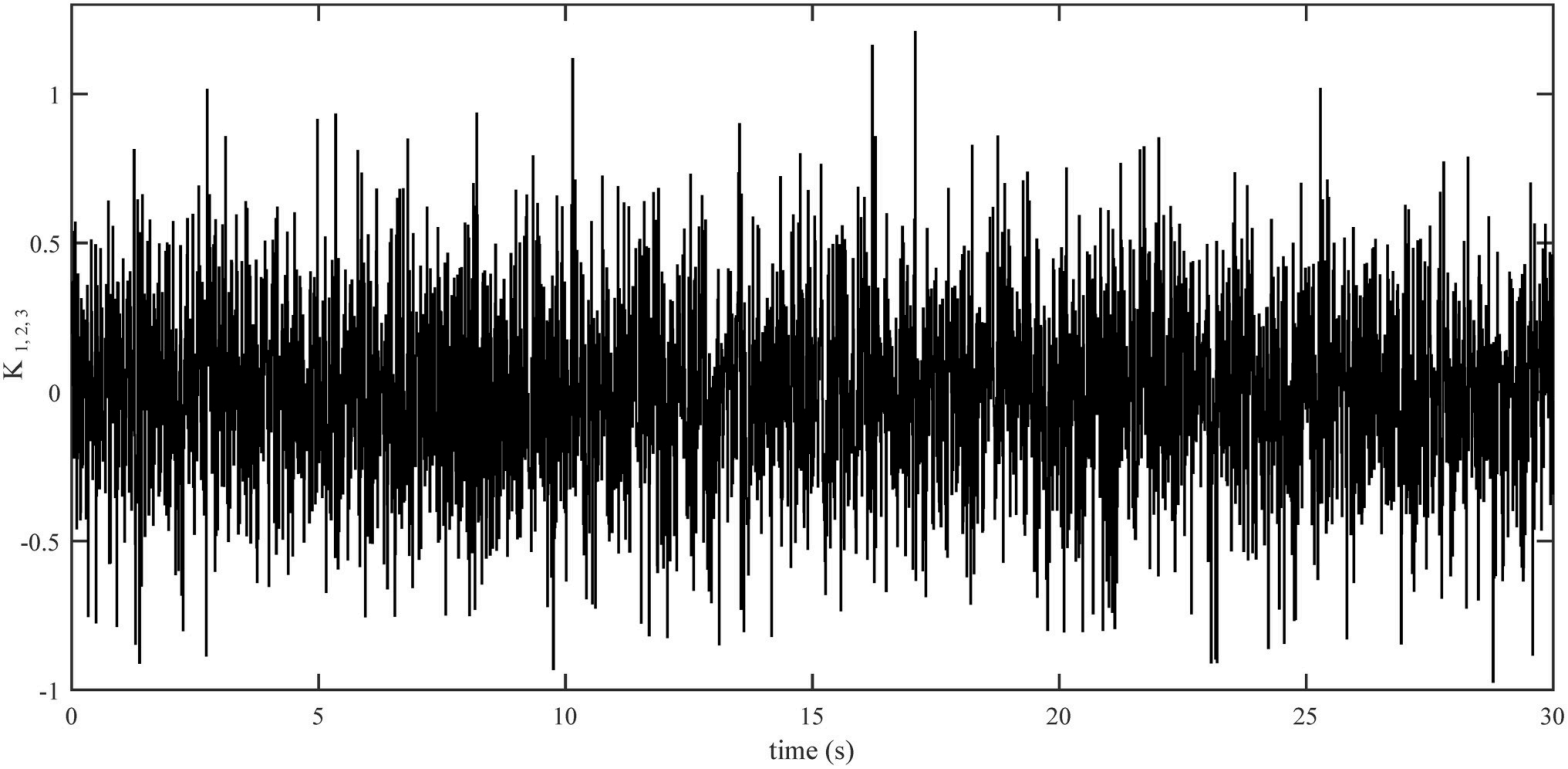
Drag coefficients for rotational motion.

The drag coefficients are supposed to change in the form of Band-Limited White Noise in a limited interval in this simulation. Obviously, the shifting curves of drag coefficients in a real fly in an environment are not more intricate than the ones presented in Figs 12 and 13.

**Fig 14:**
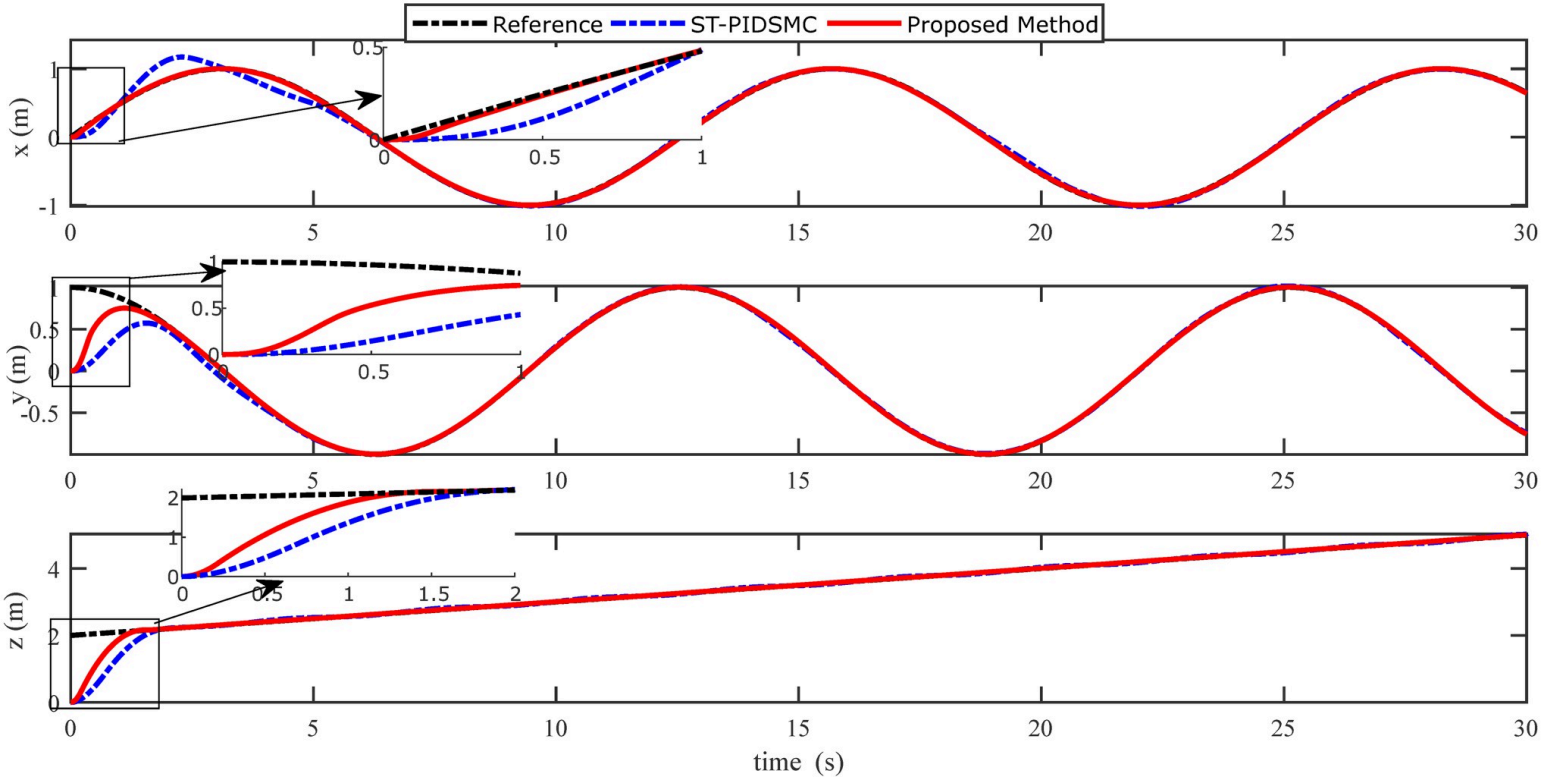
Results of the position under the proposed and ST-PID-SM controllers.

**Fig 15:**
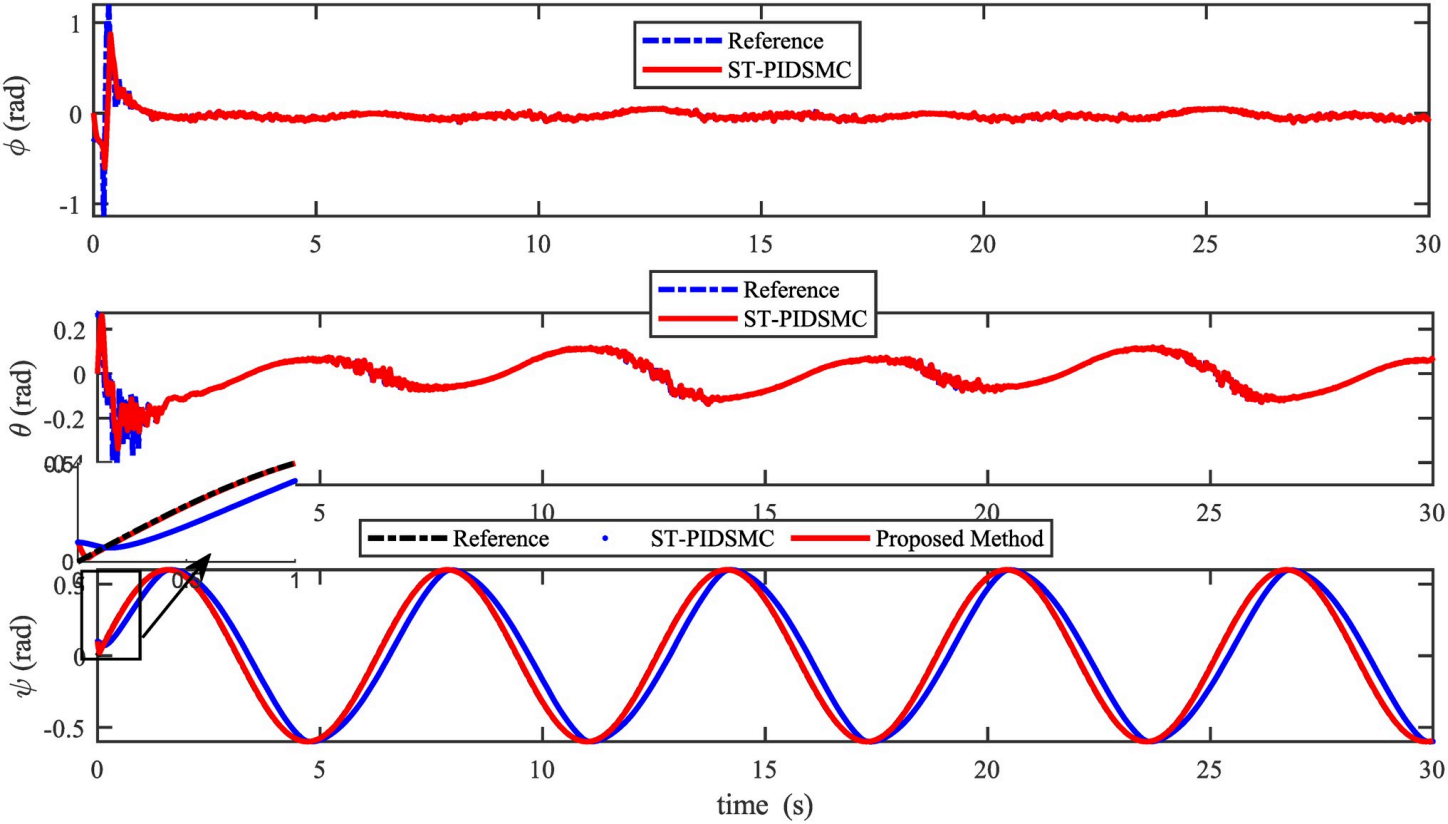
Results of the attitude under the proposed and ST-PID-SM controllers.

**Fig 16:**
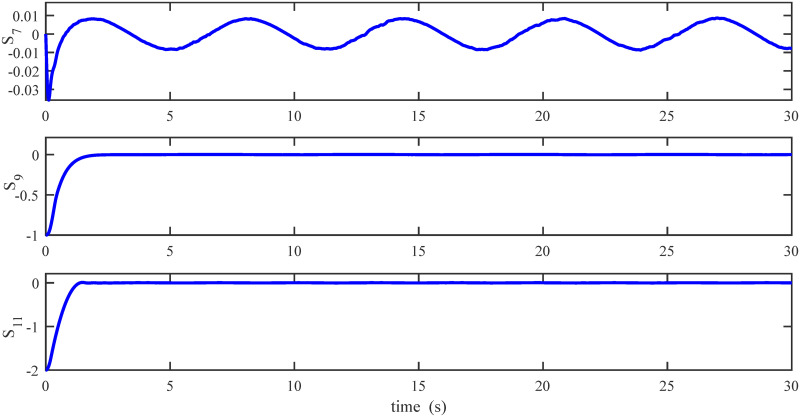
Results of the position sliding variables under the proposed controller.

**Fig 17:**
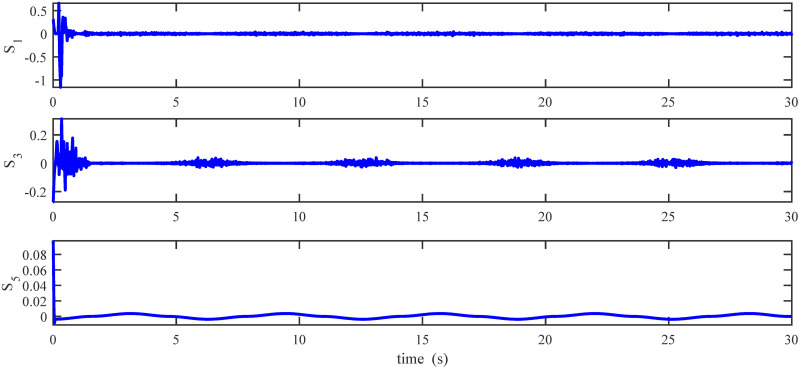
Results of the attitude sliding variables under the proposed controller.

**Fig 18:**
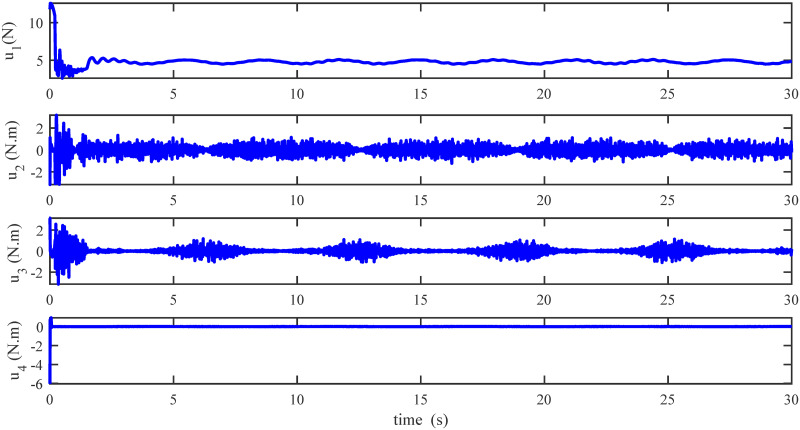
Results of the inputs under the proposed controller.

**Fig 19:**
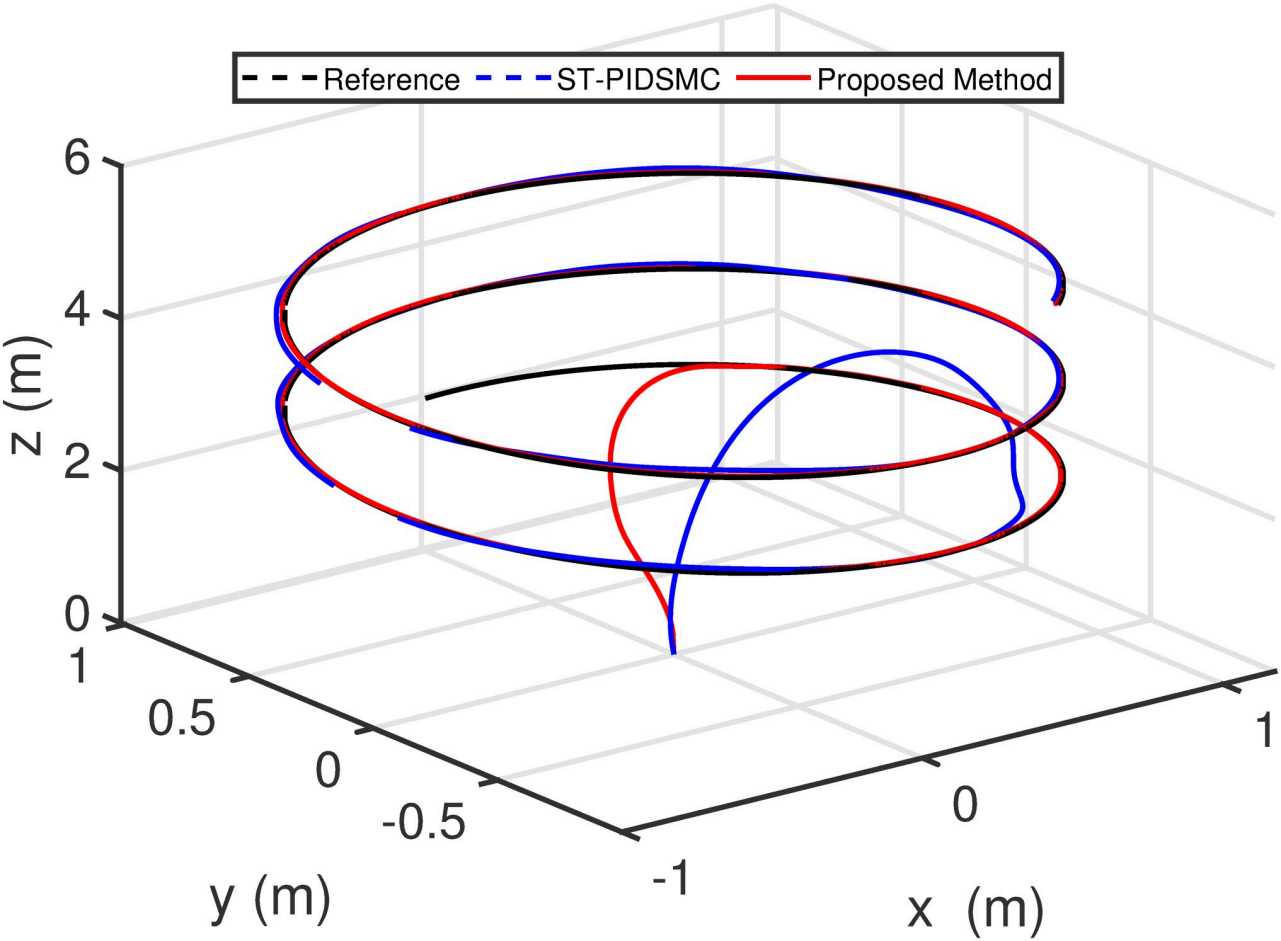
Results of the path following in 2D space under the proposed controller.

**Fig 20:**
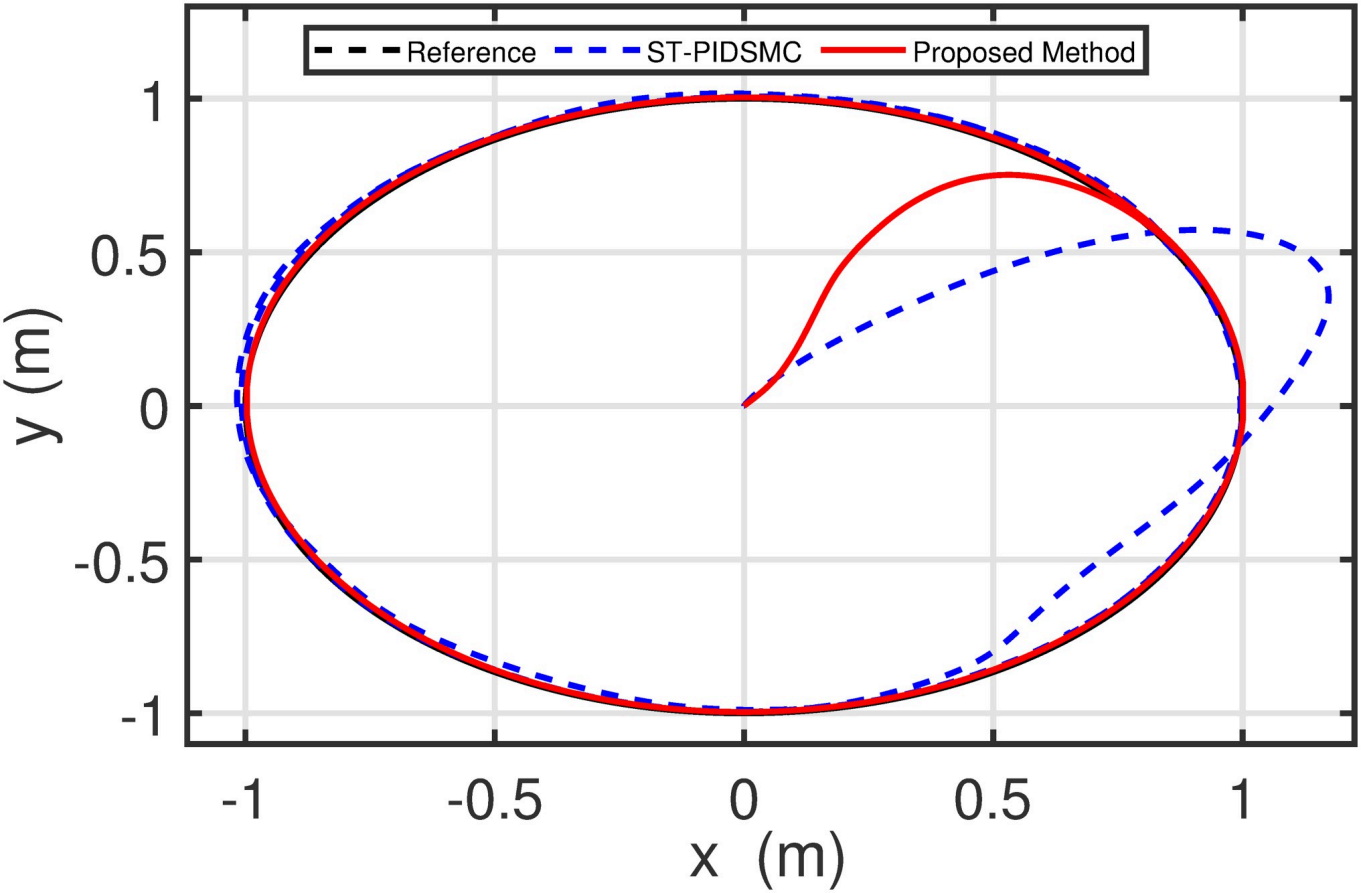
Results of the path following in 3D space under the proposed controller.

The initial conditions of quadrotor outputs in this case are zero. It should be emphasized that in this simulation,for all flight periods, the external disturbances were applied to the dynamic system to see if the proposed controller could reject the external disturbance and settle down. The outputs of a quadrotor are commended for tracking the following desired trajectory. The results tracking profiles in this simulation under the disturbances are shown in Figs 14–20. It is clear that the proposed control scheme can effectively resist external disturbances and achieve steady-state behavior. Despite this external disturbance, the hyperplane-based sliding mode control strategy provides good responses. The performance of both controllers is assessed by computing their tracking performance in 3D and 2D spaces, which are presented respectively in Figs 19 and 20, in order to clearly show the benefit.Compared to the ST-PID-SMC, the suggested controller significantly reduces the tracking error caused by external disturbances. Furthermore, the suggested controller’s control signals are substantial and have smaller values (see Fig 18). On the other hand, as shown in Figs 16 and 17, the sliding variables converge to zero in short finite-time. As a result, the suggested control method proposed in this paper for a disturbed quadrotor system has a high level of robustness against time-varying disturbances compared to the results provided by the Ref. [17].

**Remark 2**. *The dynamic model involves forces and torques applied to the quadcopter as the control actions, in order to achieve the desired reference while taking into account the inertial properties of the quadcopter. The propulsion control system, together with the servomotors that move various elements, such as the flaps of fixed-wing drones or the swashplate of helicopters, constitute the low-level control. The low-level dynamics, formulated using a first order transfer function of the system, then the dynamics of this part are very fast compared to the quadcopter dynamics. In this research, complex random parametric uncertainties and external disturbances are taken into account in two scenarios in order to make the simulation more realistic*.

### 4.4 Quantitative analysis of the controllers

The integral of the error square (ISE) and integral absolute error (IAE) are used for quantitative comparison. The ISE and IAE are numerical representations of tracking-error performance.

The ISE and IAE performances of two controllers for the scenario 1 is shown in Tables 3 and 4. Also, Tables 5 and 6 show the ISE and IAE performances for the scenario 2. In comparison to ST-PID-SMC, the finite-time control shows that the ISE and IAE indices are less important for both scenarios.

**Table 3:** ISE performance indexes of the scenario 1.

Variable	Proposed method	ST-PID-SMC
*x*(*t*)	0.0614	0.1249
*y*(*t*)	0.3665	0.781
*z*(*t*)	0.2703	0.5739
*ψ*(*t*)	0.0028	0.0225

**Table 4:** IAE performance indexes of the scenario 1.

Variable	Proposed method	ST-PID-SMC
*x*(*t*)	0.3979	0.6126
*y*(*t*)	0.6902	1.87
*z*(*t*)	0.5382	1.017
*ψ*(*t*)	0.0295	0.1349

**Table 5:** ISE performance indexes of the scenario 2.

Variable	Proposed method	ST-PID-SMC
*x*(*t*)	0.0012	0.13
*y*(*t*)	0.358	0.72
*z*(*t*)	1.46	2.53
*ψ*(*t*)	0.0002	0.323

**Table 6:** IAE performance indexes of the scenario 2.

Variable	Proposed method	ST-PID-SMC
*x*(*t*)	0.164	1.164
*y*(*t*)	0.63	1.35
*z*(*t*)	1.22	2.34
*ψ*(*t*)	0.0581	2.66

The superior tracking control performance of the proposed finite-time method is confirmed. It provides more accurate tracking, a faster convergence rate, and excellent robustness than ST-PID-SMC technique.

Compared to the results of the other approaches, the ISE and IAE values for the tracking errors are lower. All of these findings show that the proposed control method achieves better tracking performance, including high precision tracking, quick response, smooth control commands, and high robustness.

**Remark 3**. *The proposed control method has been compared to ST-PID-SMC technique, which deals with the tracking problem of the quadrotor system subject to external disturbances and parametric uncertainties. Also, in contrast to ST-PID-SMC, where the tracking errors can only asymptotically converge, the proposed control scheme, using a terminal sliding surface manifold, can guarantee the system’s finite-time zero-error stability, resulting in better steady-state performance*.

**Fig 21:**
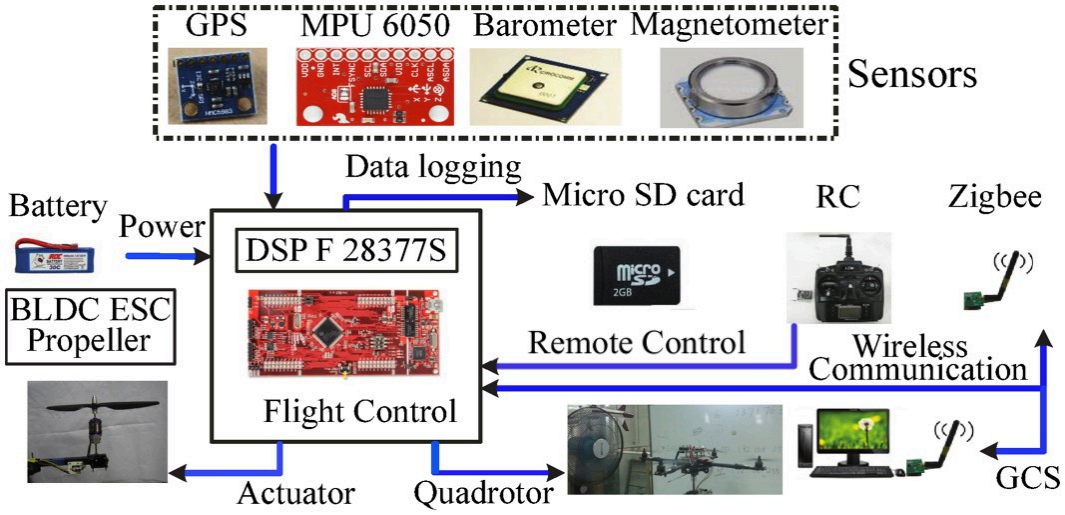
The quadrotor’s hardware structure.

**Remark 4**. *This note goes at describing the procedure for experimental validation of the finite-time control scheme. In this context, to build the quadrotor test bench experimental to validate the proposed scenarios, a list of the pieces of equipment has been compiled. The hardware configuration of the quadrotor experimental platform is depicted in* Fig 21. *This platform uses a quadrotor of version X450 with one ground control station, DSP TMS320F28379D, Inertial Measurement Unit, the global positioning system module measures the velocity and position in the horizontal plane, and barometric sensor measure the altitude. Moreover, two Zigbee wireless modules ensure the communication between the quadrotor and the ground station. A fan generates the wind then applied as disturbances to the quadrotor*.

**Remark 5**. *The present work presents a finite-time controller for a quadrotor under disturbances using a hyper-plan sliding mode manifold. Also, a switching finite-time is proposed for the system to ensure finite-time stability and cope with the upper bound of the disturbances. Moreover, in the next step, we design the proposed control method’s observer or adaptive version*.

## 5 Conclusions

This paper was devoted to the path following a quadrotor system subject to external disturbances. To begin, the new sliding manifolds for quadrotor attitude and position incorporate two variables of nonlinear sliding surfaces: nonsingular terminal sliding mode and integral terminal sliding mode. The developed sliding manifolds ensured a faster rate of quadrotor state convergence. Second, the switching control laws are built to deal with the most severe wind disturbances. The Lyapunov theory was used to verify the finite-time stability of the proposed control strategy, which improved the tracking performance of a quadrotor control system against wind disturbances. In comparison to supper-twisting PID sliding mode controller, the results obtained and the Table 1 show that the control approach suggested in this work has good tracking accuracy, convergence rate, and resilience against wind disturbances.

For further work, the finite-time approach will be validated by experiment. Design a fractional-order finite-time control technique to improve the performances of the proposed control method. Also, the fault-tolerant control problem of the quadrotor actuators and sensors will be addressed using the adaptive version of the proposed finite-time controller. Innovative solutions and sensors have recently been created for civilian use, thanks to new technologies, allowing for more flexibility (fewer restrictions in terms of sensor installation), performance (longer duration, better aerodynamic profile, better navigation system), and planning tools. The development of low-cost flight controller systems and the widespread dissemination of structure from motion applications, which allow the production of a 3D model from a sequence of photos collected from various points of view, are the most recent advancements in quadrotor aircrafts.
